# ﻿Unveiling an asymmetric plant–fungal symbiosis: morphological, cytogenetic, and molecular characterization of a haploid *Epichloë festucae* strain associated with three polyploid cytotypes of the Iberian endemic grass *Festuca rothmaleri*

**DOI:** 10.3897/imafungus.16.162692

**Published:** 2025-10-24

**Authors:** Alba Sotomayor-Alge, Luis A. Inda, Ernesto Ángel-Beamonte, Íñigo Zabalgogeazcoa, Pilar Catalán

**Affiliations:** 1 Department of Agricultural and Environmental Sciences, High Polytechnic School of Huesca, University of Zaragoza, Carretera de Cuarte s/n, 22071 Huesca, Spain University of Zaragoza Huesca Spain; 2 Biochemistry, Biophysics and Computational Biology Group, Institute for Biocomputation and Physics of Complex Systems (BIFI), University of Zaragoza – Associated Unit to CSIC, Edificio I+D, C/ Mariano Esquillor s/n, 50018 Zaragoza, Spain University of Zaragoza – Associated Unit to CSIC Zaragoza Spain; 3 Agri-Food Institute of Aragón (IA2), Agrifood Research and Technology Centre of Aragón (CITA) – University of Zaragoza, Avda. de Montañana 930, 50059 Zaragoza, Spain Agrifood Research and Technology Centre of Aragón (CITA) – University of Zaragoza Zaragoza Spain; 4 Institute of Natural Resources and Agrobiology of Salamanca (IRNASA), Spanish National Research Council (CSIC), C/ Cordel de Merinas 40–52, 37008 Salamanca, Spain Institute of Natural Resources and Agrobiology of Salamanca (IRNASA), Spanish National Research Council (CSIC) Salamanca Spain

**Keywords:** Asymmetric symbiosis, *
Epichloë
*, *
Festuca
*, flow cytometry, fungal endophytes, morphometrics, phylogenetics, polyploidy

## Abstract

The ecological and evolutionary outcomes of plant–fungal interactions are strongly influenced by genome size and ploidy, yet the ploidy level of both partners is rarely assessed simultaneously. *Epichloë* symbioses with *Pooideae* grasses are established model systems for exploring these dynamics, but associations between polyploid hosts and haploid endophytes remain poorly documented. In this study, the association of the Iberian endemic *Festuca rothmaleri*—which includes tetraploid, hexaploid, and octoploid cytotypes—with *Epichloë* fungal endophytes is documented for the first time. An integrative, method-rich framework combining cytogenetics, morphometrics, and multilocus phylogenetics revealed a strikingly asymmetric interaction, with all cytotypes harboring a single haploid strain of *Epichloë festucae*. Two methodological innovations were developed: (i) an image-based tool for automated measurement of asexual structures, including the novel metric “conidial area,” and (ii) a flow cytometry protocol for estimating fungal genome size. Despite morphological variability, all fungal isolates shared similar genome sizes and formed a well-supported monophyletic lineage in a coalescent species tree based on nuclear loci sequences (*actG*, *CalM*, ITS, *tefA*, *tubB*). This work provides the first comprehensive characterization of a haploid *Epichloë* endophyte spanning multiple naturally distributed host ploidy levels and highlights a rare but promising system for future evolutionary, physiological, and ecological studies of plant–fungal interactions.

## ﻿Introduction

Plant–fungal symbioses are among the most ecologically and evolutionarily significant interactions in terrestrial ecosystems, with an estimated one-third of true fungi engaging in finely tuned associations with plants (Kendrick 1991). Among other factors, ploidy—an important aspect of genomic architecture—has been shown to influence these relationships. Host ploidy variation can alter infection frequency, vertical transmission, and physiological processes (e.g., Gundel et al. 2012; Vázquez de Aldana et al. 2013), while heteroploidization in fungal endophytes can broaden host range (Moon et al. 2004) and diversify alkaloid biosynthesis (Schardl et al. 2013). Despite these insights, the ploidy of both partners in the interaction is rarely assessed simultaneously and its role in shaping compatibility and co-adaptation remains poorly explored. Within this context, the symbiotic associations between cool-season grasses (*Poaceae*, *Pooideae*) and endophytic fungi of the genus *Epichloë* Fr. (*Clavicipitaceae*, *Ascomycota*; Leuchtmann et al. 2014) are among the most intensively studied plant–microbe interactions and serve as a model system to assess the evolutionary, physiological, and ecological dynamics of symbiosis (Saikkonen et al. 2016). These interactions are widespread and diverse, with outcomes ranging from mutualism to antagonism, and depend on factors such as the onset of the endophyte’s sexual cycle or hyphal growth regulation (Scott et al. 2018). Beyond host performance, endophyte-mediated trait shifts can cascade to changes in plant community composition, herbivore interactions, and ecosystem processes, highlighting their broader ecological relevance (e.g., Saikkonen et al. 2016; Xia et al. 2018). The prevailing evidence supports its role as a “defensive mutualism” (Clay 1988), in which the endophyte gains nutritional, reproductive, and dispersal advantages, while the host enhances its tolerance to abiotic (e.g., drought, salinity; Wang et al. 2020) and biotic stresses (e.g., herbivory, pathogens; Xia et al. 2018; Hewitt et al. 2021) through protective alkaloids produced by the fungal partner (Schardl et al. 2013). Moreover, this interaction has far-reaching physiological and agroeconomic implications, underpinning its use in pasture and turf systems to enhance persistence, productivity, and resistance to grazing and pests; thereby contributing to more sustainable and resilient grass-based agriculture (e.g., Cagnano et al. 2019; Caradus and Johnson 2020).

*Epichloë* fungal endophytes are biotrophic symbionts characterized by host-specific interactions, systemic colonization of aerial tissues, and a marked propensity for hybridization (Schardl 1996; Clay and Schardl 2002; Moon et al. 2004; Gentile et al. 2005; Leuchtmann and Schardl 2005). They inhabit the apoplast of their hosts, reproducing sexually, asexually, or through mixed life cycles. Asexual reproduction occurs via vertical transmission through host seeds, whereas sexual reproduction entails stromata formation, ascospores production, and horizontal transmission at the expense of host inflorescences—a phenomenon known as “choke disease” (Leuchtmann et al. 2014; Tadych et al. 2014). According to Moon et al. (2004) and Tadych et al. (2014), sexual reproduction is largely confined to haploid, non-hybrid species, whereas asexual transmission predominates in haploid and hybrid heteroploid taxa, with only a few exceptions (Yan et al. 2011).

The genus *Festuca* L., within the subtribe *Loliinae*, is among the most diverse and ecologically important grass genera. It is distributed worldwide and comprises more than 600 species (Catalán 2006; Moreno-Aguilar et al. 2022; Plants of the World Online [https://powo.science.kew.org/taxon/urn:lsid:ipni.org:names:328907-2], accessed September 30, 2025), including many major fo­rage, pasture, and lawn grasses (Catalán 2006; Kopecký and Studer 2014). The complex phylogenetic relationships among these species have been extensively studied (Catalán et al. 2004; Inda et al. 2008; Šmarda et al. 2008; Cheng et al. 2016; Minaya et al. 2017; Moreno-Aguilar et al. 2020, 2022), and recent analyses support the divergence of two major fine-leaved (FL) and broad-leaved (BL) *Loliinae* clades, as well as up to 29 globally distributed sublineages (Moreno-Aguilar et al. 2024).

The *Festuca*–*Epichloë* interactions have been extensively studied, leading to the identification and characterization of isolated *Epichloë* strains, but rarely examining the plant host in depth (Suppl. material 1: table S1). Methodologies applied include macroscopic and microscopic morphology (Leuchtmann 1994; Scott et al. 2012; Tian et al. 2020), mating compatibility (Schardl and Leuchtmann 1999; Chen et al. 2015), chemotypic diversity (Saikkonen et al. 2016; Soto-Barajas et al. 2019), and molecular approaches ranging from barcoding markers such as elongation factor 1-α and β-tubulin (Moon et al. 2002; Gentile et al. 2005) to whole-genome sequencing (Winter et al. 2018; Thünen et al. 2022). Among these endophytes, *E. festucaesensu lato* (s.l.) is the most widespread symbiont across the genus, reported primarily in fine-leaved *Loliinae* taxa (e.g., Moon et al. 2004; Zabalgogeazcoa et al. 2006a; Gibert and Hazard 2011; Vázquez De Aldana et al. 2015; von Cräutlein et al. 2021) and more rarely in broad-leaved *Loliinae* (Bush et al. 1997; Cagnano et al. 2019) and intermediate *Loliinae* lineages (Leuchtmann et al. 1994; Niones and Takemoto 2014). Some studies have also reported *E. festucae* in polyploid *Festuca* species, including *F. rubra* s.l. (tetraploid, hexaploid, and octoploid cytotypes; Dirihan et al. 2016) and *F. vivipara* (tetraploid; Gundel et al. 2014), but without a direct examination of the genome size or ploidy of the fungal partner (Suppl. material 1: table S1). Furthermore, documented cases such as *E. coenophiala* in the allohexaploid grass *F. arundinacea* (Tsai et al. 1994) indicate that hybrid or polyploid hosts can influence endophyte diversity and may facilitate hybridization events. Phylogenetic analyses of nuclear loci (Schardl et al. 1994; Moon et al. 2004) have also suggested that the hybrid origins of some heteroploid *Epichloë* endophytes parallel the allopolyploid nature of their hosts, implying that host hybridization and subsequent somatic fusion (Shoji et al. 2015) may drive the formation of multi-genome endophytes. Hybrid endophytes have also been described in diploid hosts, such as *E. hybrida* in *Lolium perenne* (Moon et al. 2004), revealing that genomic complexity can arise in the fungal partner even when the host is not polyploid. Nevertheless, few studies have examined the potential correlation between ploidy levels and genome size in *Festuca* hosts and their *Epichloë* endophytes (Suppl. material 1: table S1). This gap is particularly relevant when a single haploid endophyte colonizes the same host species with different ploidy levels, offering a natural framework to study how symmetry in genome content and ploidy is tolerated or maintained, and the extent of its ecological and evolutionary consequences (Schardl et al. 2004; Bonfante and Genre 2010).

To address this matter, this work presents the first comprehensive characterization of a natural system in which a single haploid *E. festucae* strain colonizes three different cytotypes of the *F. rothmaleri* (Litard.) Markgr.-Dannenb. polyploid complex. *F. rothmaleri* is endemic to the northwestern Iberian Peninsula and is characteristic of mountain and dehesa pastures (Loureiro et al. 2007). It displays distinctive morphoanatomical features distinguishing it from related species, as well as notable phenotypic plasticity (De la Fuente and Sánchez 1987) and cytotypic diversity, with tetraploid, hexaploid, and octoploid populations reported (Saikkonen et al. 2019; Devesa et al. 2020). Phylogenetically, it belongs to the *Aulaxyper* lineage (*F. rubra* group) within the fine-leaved *Loliinae* clade (Inda et al. 2008). Previous surveys detected *Epichloë* endophytes in populations of the *F. rubra* complex across Europe (Zabalgogeazcoa et al. 1999; Vázquez De Aldana et al. 2015), but the specific host taxa involved were not identified at species level.

Hence, a multi-method approach was applied to characterize the interaction between *F. rothmaleri* cytotypes and a single haploid strain of *E. festucae*, assessing: (a) endophyte incidence; (b) mating-type composition of the endophyte as a proxy for sexual potential; (c) morphology of diagnostic structures in both sym­bionts; (d) cytogenetic features of both symbionts; and (e) multilocus phylogenetic characterization of the endophyte. Moreover, two methodological advances were developed: a digital image–based software to automatically measure conidial traits (e.g., conidial area) and the first application of flow cytometry to estimate *Epichloë* genome size. Together, these efforts provide the first detailed description of this endemic grass–endophyte system and establish a new platform to further explore how ploidy asymmetry shapes ecological and evolutionary dynamics.

## ﻿Methods

### ﻿Sampling and characterization of host plants

Sampling and taxonomic identification

Individuals of *Festuca rothmaleri* representing three putative ploidy levels (tetra­ploid, hexaploid, and octoploid) were collected from three mountainous sites in northwestern Spain, Montemayor del Río (Mon), Candelario (Can), and El Cabaco (Cab) (Salamanca province), located 20–50 km apart and separated by mountain ranges (Suppl. material 1: fig. S1). Between 20 and 35 individuals were sampled per site during two consecutive flowering seasons (June 2022 and June 2023). To reduce the likelihood of clonal sampling due to the rhizomatous growth habit of the species, individuals were collected at a minimum distance of 3 meters from one another. Plants were transplanted into pots with universal substrate (Blumenerde, Gramoflor) and maintained in a greenhouse under relatively constant temperature conditions (22–27 °C) and watering regimes (adjusted according to seasonal needs, on average three times per week) throughout the study.

Taxonomic identification of the host plants was performed based on morpho­anatomical examination and measurements of vegetative and reproductive structures, following the diagnostic criteria and identification keys provided by De la Fuente and Sánchez (1987), Al-Bermani et al. (1992), De la Fuente et al. (2001), Loureiro et al. (2007), and Devesa et al. (2020).

#### ﻿Cytogenetic characterization

The host plant genome size was determined by flow cytometry (Ploidy Analyzer, Sysmex) in fresh mature leaf tissue, following the protocol and reagents (Otto I and Otto II) described by Doležel et al. (2007). A preliminary analysis confirmed the existence of three genome size categories corresponding to different ploidy levels. Based on these results, up to 15 individuals were selected per population of origin and ploidy level (Mon4x, Can4x, Cab6x, and Can8x), analyzing two technical replicates in each case. Measurements were made on more than 5,000 nuclei with a coefficient of variation (CV) < 3%. Primary standards included *Solanum lycopersicum* ‘Stupicképolnírané’ (1.96 pg/2C) for tetraploids, *Pisum sativum* ‘Ctirad’ (9.09 pg/2C) for octoploids, and *Secale cereale* ‘Daňkovské’ (16.19 pg/2C) for hexaploids according to Doležel et al. (2007).

Chromosome number was determined from meristematic cells of the root tip using the protocol of Jenkins and Hasterok (2007). For each ploidy level, five independent counts were performed for each of three randomly selected individuals from the genome-size dataset, resulting in a total of 15 counts per detected ploidy level. Images were obtained at 400× magnification using a Zeiss Axio Lab.A1 phase-contrast microscope equipped with a Canon EOS 2000D digital camera.

### ﻿Comprehensive multi-method characterization of *Epichloë* endophytes

#### ﻿Detection, isolation and incidence

The presence of the endophyte in the host’s aerial tissues was initially detected by aniline blue staining (Florea et al. 2015). In parallel, fragments of floral stems or the bases of vegetative tillers from all collected specimens, with the surface disinfected, were cultured on potato dextrose agar (PDA, Potato Dextrose Agar EP/USP/BAM, Condalab) plates containing chloramphenicol (Chloramphenicol BioChemica, PanReacAmpliChem; 25 µg/ml) to inhibit bacterial growth. When an *Epichloë*-like endophyte emerged from the host tissue, it was isolated on PDA plates and cultured at room temperature (22–25 °C) in the dark. The incidence of *Epichloë* endophytes in *Festuca* hosts varies by holobiont and locality (Zabalgogeazcoa et al. 1999; Saikkonen et al. 2000; Clement et al. 2001; Bazely et al. 2007; Gundel et al. 2014). Thus, the proportion of infected individuals was estimated by sampling site (Mon, Can, Cab), host ploidy level (4x, 6x, 8x), and their combination (Mon4x, Can4x, Cab6x, Can8x), considering both detection methodologies.

#### ﻿Exploratory mating-type composition screening

During field collections, no stromata were observed in *F. rothmaleri* individuals. Therefore, the sexual reproduction potential of the endophyte was evaluated through exploratory PCR screening for the MAT1-1 (785 bp) and MAT1-2 (215 bp) idiomorphs (Florea et al. 2015) in 4–6 *F. rothmaleri* individuals per sampling site (Mon, Cab, Can). Sample sizes were adjusted to ensure detection of both idiomorphs at each location, using total DNA extracted from leaf tissue. Primer sequences and PCR conditions are provided in Suppl. material 1: table S2.

#### ﻿Morphological analyses and growth rate

Asexual reproductive structures (conidia and conidiophores) have proven to be a valuable taxonomical trait for the identification of *Epichloë* endophytes (Gentile et al. 2005; Leuchtmann and Schardl 2005; McCargo et al. 2014; Campbell et al. 2017; Thünen et al. 2022). The dimensions of these structures have also been correlated with the putative ploidy level of the endophytes (Kuldau et al. 1999). To assess the natural variability in these traits and their potential link to ploidy, four *Epichloë* isolates from hosts with differing source population and ploidy levels (i.e., Mon4x, Can4x, Cab6x, Can8x) were analyzed, with three biological replicates per isolate. Microscopic preparations consisted of 1 mm² sections of mycelium, grown for three weeks on water agar (WA; European Bacteriological Agar, Condalab), which were carefully melted and sealed under a coverslip. The length and width of 10 conidia, along with the total length and width at the base of 10 conidiophores, were recorded for each replicate, yielding a total of 30 entries per isolate. To morphometrically characterize these endophytes, a novel software –*Epichloë* conidia– was developed using Matlab version 9.3 (R2017b; see Suppl. material 1: appendix S1) to automatically detect and estimate conidial area. Only spores photographed in sagittal plane were analyzed to ensure comparability, using both ImageJ v154g (Schneider et al. 2012) and custom software, with a fixed scale of 15.8097 pixels/μm. Morphometric differences among groups were assessed using PERMANOVA based on Euclidean distances with 10,000 permutations (R package ‘vegan’ v2.6.10; Oksanen et al. 2025), which does not assume multivariate normality and is suitable for nested designs. Pairwise permutation tests (R package ‘coin’ v1.4.3, Hothorn et al. 2008) were also performed with 10,000 resamples, considering the clustering structure of the study: sampling sites (n = 3), host ploidy levels (n = 3), individuals per source population and host ploidy level (n = 4), and replicates within individuals (n = 10). *P*-values were adjusted using the Holm-Bonferroni method, with significance set at p < 0.05.

Additionally, exploratory multivariate analyses, including Principal Component Analysis (PCA, R package ‘FactoMineR’ v2.11, Lê et al. 2008) and Linear Discriminant Analysis (LDA, R package ‘MASS’ v7.3.60.2), were carried out to identify potential patterns of endophyte diversification associated with population origin, host ploidy level, or their interaction. These analyses were performed under three data clustering schemes: (i) individual-level mean-pooled measurements (n = 16); (ii) replicate-level mean-pooled measurements (n = 48); and (iii) resampling-level individual measurements (n = 469). This hierarchical structuring of sample sizes was designed to help identify the main sources of variability within the system. To statistically evaluate the morphological variation revealed by PCA, linear models (LM) and analyses of variance (ANOVA) were developed on the scores of the first two principal components (PC1 and PC2), including host ploidy level, population of origin, and their interaction as explanatory variables, using the R package “stats” v4.4.1. The performance of the LDA models was assessed using cross-validation procedures implemented with the R package “caret” v6.0.94 (Kuhn 2008).

Since the growth rate of fungal cultures on PDA medium has been used as a trait to characterize *Epichloë* species, 1.5 mm^2^ sections of four two-week-old isolates per population were simultaneously grown on PDA plates at room temperature in the dark for 24 days to estimate endophyte growth rate. Three technical replicates per isolate were analyzed. Culture growth was monitored every eighth day by taking pictures and measuring the diameter of the culture. Growth front was considered the external limit for this measure and a digital vernier caliper was used to record culture diameter. Growth rate (GR; mm/day) was calculated for each individual sample following the equation GR = (Diameter_t´_-Diameter_t_) / Δ_t_, where Diameter_t_ is the initial diameter of the culture, Diameter_t’_ is the final diameter and Δ_t_ is the total number of days. Mixed-effect models were fitted due to the dependency between the measurements registered (i.e., diameter measured through time) and the hierarchical structure of the experimental design using the R package ‘lme4’ v1.1.35.5 (Bates et al. 2015). The effect of the source population, host ploidy level, and their interaction (fixed effects) on fungal growth rate (mm/day) was analyzed, including individuals or replicates as random effects to account for intra-individual variability and make more precise estimates without inflating standard errors. ANOVAs and subsequent pos-hoc pairwise tests assessing differences by marginal means (EMMs) applying Tukey’s HSD correction were carried out to test the significance of these models and detect potential sources of variability using the R package ‘emmeans’ v1.10.5 (Lenth 2024). All these analyses were performed in R v4.4.1 (R Core Team 2024) using a custom script (script1.Rmd) created with R Markdown (rmarkdown’ v2.29; Allaire et al. 2024).

#### ﻿Genome size estimation

DAPI-stained slides were prepared to confirm that the endophyte conidia were uninucleate before performing genome size estimations. Two-week-old 1 mm^2^ slices of mycelium were grown on a nutrient-deficient medium, consisting of water agar, for 15 days at room temperature and in the dark to facilitate sporulation. Photographs were taken with a fluorescence microscope (Miotic BA410) equipped with the Miotic MoticamPro 285D camera.

Existing protocols for other fungal species were adapted to the endophytes under study by flow cytometry (Ploidy Analyzer, Sysmex) to determine their genome sizes and infer the ploidy levels of the endophytes isolated from the different *F. rothmaleri* cytotypes. *Colletotrichum acutatum* strain PT812 (68 Mb or ~ 0.069 pg/1C) was included as primary standard following Talhinhas et al. (2017). Each *Epichloë* isolate and the standard were ground with a sterile mortar and pestle in 700 µl of ddH_2_O and grown for 7–10 days in PDA plates with a sterile cellophane disk to increase the availability of active young mycelia. A 0.5 cm^2^ portion was extracted and grounded in a glass petri dish with 1 ml of LB01 buffer (Doležel et al. 2007) using a sharp blade. The nuclear suspension was filtered through a 20 μm nylon mesh filter before being stained with 50 μl of propidium iodide. After a brief incubation on ice (3–5 minutes), samples were analyzed using the Ploidy Analyzer flow cytometer (Sysmex). Sample processing was carried out on ice (~ 4 °C) to ensure nuclear stability. Three isolates were considered to establish the genome sizes of endophytes from each source population and the ploidy level of the host (Mon4x, Can4x, Cab6x, Can8x), measuring 4 technical replicates in each case (n = 12). Here, a minimum threshold of 5000 nuclei was set for each measurement, and coefficients of variation (CV) were considered acceptable when they were below 10%, following previous flow cytometry analysis standards in other fungi (Bourne et al. 2014).

#### ﻿Molecular and phylogenetic analyses

*Epichloë* isolates were grown for 7–10 days on PDA plates covered with sterile cellophane disks to facilitate mycelial recovery. DNA was extracted using a modified version of the CTAB-based DNA extraction protocol (Doyle and Doyle 1987; see Suppl. material 1: appendix S2). Quality and concentration were assessed using a Biodrop spectrophotometer (µLite) and a Qubit 3.0 fluorometer. PCR amplified products from four nuclear loci [γ-actin (*actG*), calmodulin (*CalM*), translation elongation factor 1-α (*tefA*), and β-tubulin (*tubB*)], as well as the ITS region of rDNA, were subjected to bidirectional Sanger sequencing (Moon et al. 2004; McCargo et al. 2014; Chen et al. 2019; Thünen et al. 2022; Wang et al. 2022), using optimized primers and cycling programs detailed in Suppl. material 1: table S2. Two isolates per combination of source population and host ploidy level (Mon4x, Can4x, Cab6x, Can8x) were selected for molecular characterization.

Sequences were trimmed and aligned in Geneious Prime version 2024.0.5 (Biomatters Ltd, New Zealand) using MAFFT algorithm v7.490 (Katoh and Standley 2013) and subsequently processed manually. Single nucleotide polymorphisms (SNPs) calling was performed with a threshold value of a minimum variant frequency of 25% due to the small sample size of our dataset. Reference sequences for the five studied loci were obtained from published *Epichloë* genomes and two *Claviceps purpurea* strains that were used as outgroups (Suppl. material 1: table S3). Only haploid genomes (i.e., single-copy loci) were included, verified using a custom bash script (script2.sh). Single-gene maximum likelihood phylogenetic trees were obtained using IQ-TREE 2 (Minh et al. 2020) with 1000 UltraFast Bootstrap (BS) replicates (Hoang et al. 2017). For visualization, the lengths of all branches were transformed using x^¼^.Subsequently, both the concatenated maximum likelihood tree and a coalescence-based species tree were obtained using IQ-TREE 2 and ASTRAL-III (Zhang et al. 2018), respectively. The likelihood-based site concordance factor (sCFL) was calculated using 1000 replicates for the concatenated ML tree (Mo et al. 2023), and quartet scores for the species tree and its two alternative topologies (Sayyari and Mirarab 2016) were inferred using ASTRAL-III. All trees were generated, edited, and formatted with two custom scripts created with bash and R Markdown (script3.sh, script4.Rmd).

## ﻿Results

### ﻿Taxonomic identification and cytogenetic profile of *Festuca rothmaleri* populations

**Figure 1.**
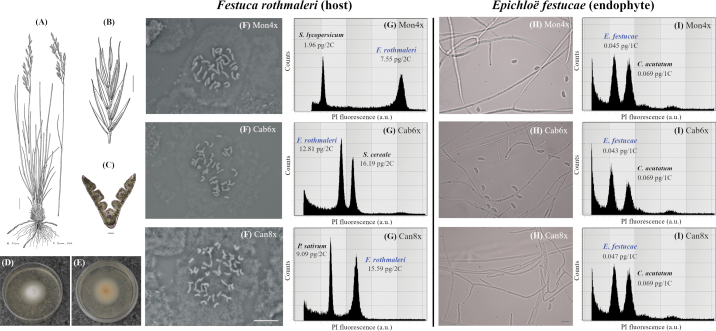
Morphological and cytogenetic diagnostic traits of *Festuca rothmaleri* and its associated *Epichloë festucae* endophytes. A–C. Habit, spikelet, and transverse leaf section of *F. rothmaleri*. D, E. *E. festucae* isolates grown on PDA (5.5 cm plates), front and reverse views respectively. F. Metaphase chromosomes of the three *F. rothmaleri* cytotypes: tetraploid (Mon4x; 2n = 4x = 28), hexaploid (Cab6x; 2n = 6x = 42), and octoploid (Can8x; 2n = 8x = 56). G. Flow cytometry histograms of nuclear DNA content for each *F. rothmaleri* cytotype (X-axis, fluorescence intensity; Y-axis, particle counts). H. Asexual reproductive structures (conidia, conidiophores) of *E. festucae* isolates from each grass cytotype. I. Flow cytometry histograms of nuclear DNA content for *E. festucae* cultures (X-axis, fluorescence intensity; Y-axis, particle counts). A, B. Reproduced with permission from Devesa et al. (2020); C. photographed at 5×. Scale bars: 2 cm (A); 2 mm (B); 10 µm (C); 5 µm (F); 5 µm (H). Genome size standards: *Solanum lycopersicum* ‘Stupicképolnírané’ (1.96 pg/2C) for tetraploid, *Secale cereale* ‘Daňkovské’ (16.19 pg/2C) for hexaploid, *Pisum sativum* ‘Ctirad’ (9.09 pg/2C) for octoploid host plants (G), and *Colletotrichum acutatum* strain PT812 (0.069 pg/1C) for *E. festucae* endophytes (I).

Sampled specimens of *Festuca rothmaleri* were morphologically studied and assigned to this taxon (Fig. 1A–C). Qualitatively, one of the best diagnostic traits of *F. rothmaleri* is its V-shaped leaf cross section with square-shaped ribs and non-scalloped abaxial surface (De la Fuente and Sánchez 1987; Devesa et al. 2020), observed in all samples studied (Fig. 1C).

**Figure 2.**
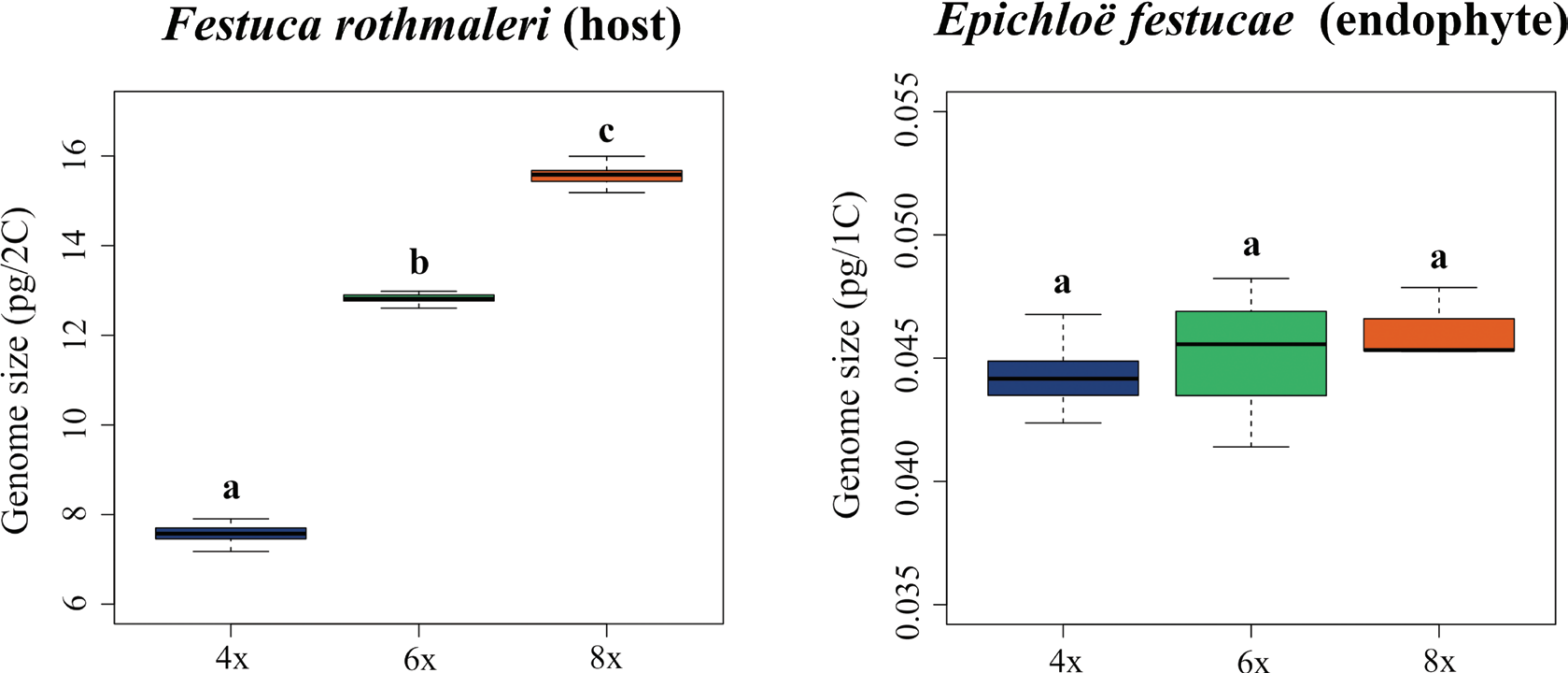
Asymmetrical genome size variation in the host *Festuca rothmaleri* (left panel) contrasts with the stable genome size of its endophyte *Epichloë festucae* (right panel) across the three polyploid cytotypes of the host. Boxplots show nuclear DNA content (Y-axis) for tetraploid (4x), hexaploid (6x), and octoploid (8x) cytotypes (X-axis) in *F. rothmaleri* individuals (pg/2C) and their respective *E. festucae* isolates (pg/1C). Both tetraploid populations (Mon4x and Can4x) showed no significant differences (ANOVA, p > 0.05) and were therefore pooled. Different letters indicate significant differences among cytotypes (ANOVA and Tukey’s HSD; p < 0.05). Whiskers indicate values within 1.5 times the interquartile range (IQR).

Cytogenetic analyses confirmed that the sampling covered all three known ploidy levels of *F. rothmaleri*. Chromosome counts and genome size estimations detected the existence of tetraploid (2n = 4x = 28; 7.54 ± 0.21 pg/2C), hexaploid (2n = 6x = 42; 12.81 ± 0.16 pg/2C) and octoploid (2n = 8x = 56; 15.58 ± 0.28 pg/2C) cytotypes (Figs 1F, G, 2; Suppl. material 1: table S4). While two of the studied populations were composed exclusively of tetraploid (Mon) or hexaploid (Cab) individuals, a third mixed population (Can) included both tetraploids and octoploids. Tetraploid individuals in the mixed Can population did not differ significantly in genome size measurements from the tetraploids in the homogenous tetraploid population Mon (ANOVA, p > 0.05; Fig. 2; Suppl. material 1: table S4).

### ﻿Characterization of *Epichloë* endophytes inhabiting *Festuca rothmaleri*

#### ﻿Incidence rates

The presence of *Epichloë* was assessed in all *F. rothmaleri* specimens collected from each population, with both detection methods yielding consistent results across all individuals. The incidence rates of this interaction ranged from relatively high (77.1% Mon, 82.4% Can) to very high levels (90.9% Cab). When considering the different host ploidy levels, the incidence rates were 81.5% among tetraploids (considering individuals from the Mon4x and Can4x populations), 90.9% among hexaploids (Cab6x), and 80.0% among octoploids (Can8x). Based on these results, this symbiont is widely distributed and likely occurs in other *F. rothmaleri* populations in the study area.

#### ﻿Exploratory mating type screening

Both MAT1-1 and MAT1-2 idiomorphs were detected across the three sampling sites (Mon, Cab and Can), with at least one endophyte genotype carrying each mating type in every site (Suppl. material 1: table S5). These findings suggest a potential for sexual reproduction among the studied *Epichloë festucae* individuals in all cases, even though no stromata have been observed to date in the studied *F. rothmaleri* populations.

#### ﻿Morphology and growth rate

The growth habit (macroscopic appearance) of the studied *Epichloë* endophytes was consistent with previous descriptions of *E. festucae* (Kuldau et al. 1999; Zabalgogeazcoa et al. 2006b; Scott et al. 2012) forming a white, fluffy mycelium with phenotypic plasticity (Fig. 1D, E, Suppl. material 1: fig. S2). Mixed-effects models for culture growth rate (GR) showed no significant effect of population, ploidy, or their interaction (all p > 0.75), indicating that growth rate was not structured by these factors. The average GR across all the studied *E. festucae* isolates was 1.69 ± 0.21 mm/day (Mean ± SD).

**Figure 3.**
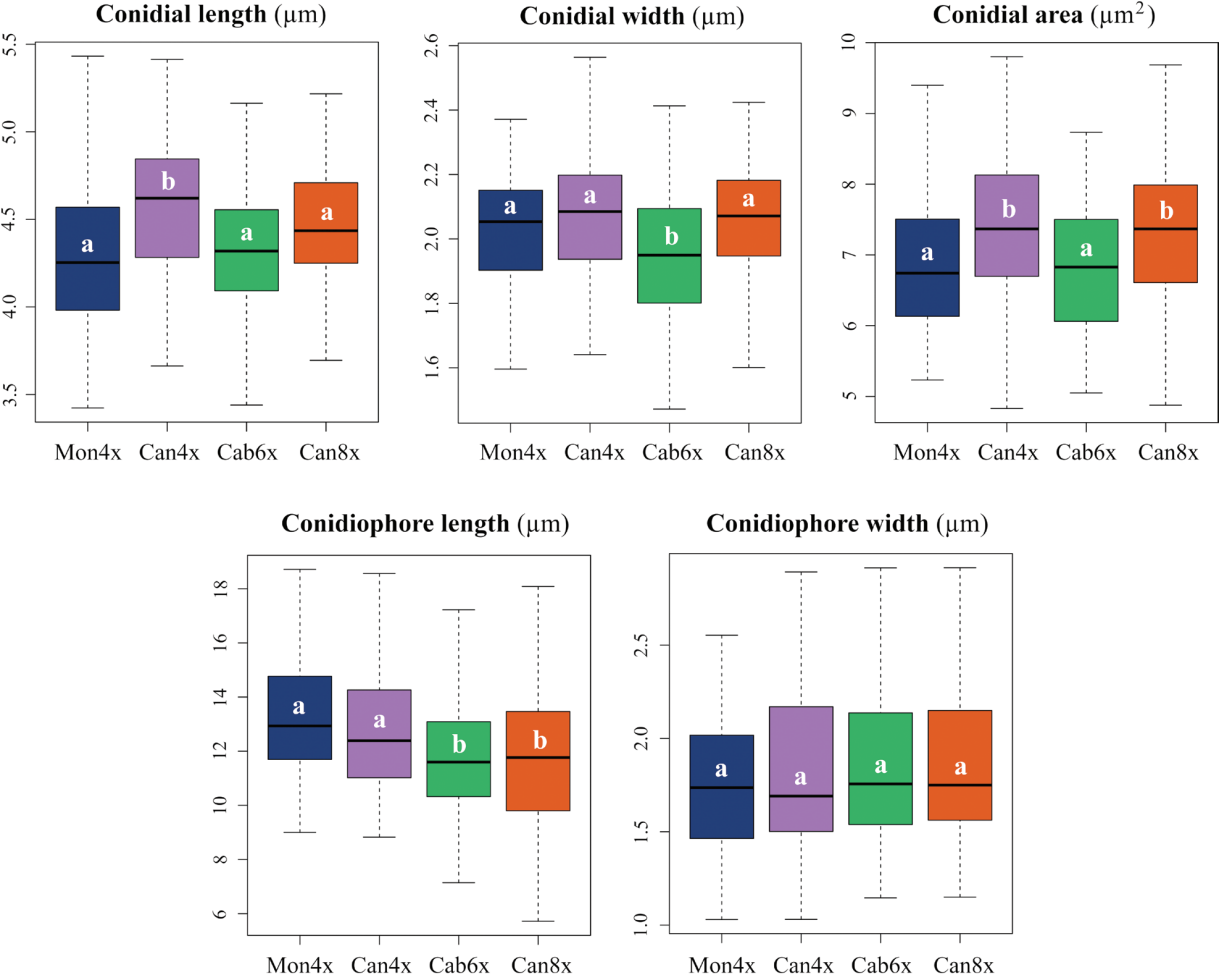
Morphometric variation in asexual structures of *Epichloë festucae* isolates from the diverse *Festuca rothmaleri* cytotypes and sampling spots (Mon4x, Can4x, Cab6x, and Can8x). Boxplots in the top panels show conidial length (µm), width (µm), and area (µm^2^); whereas bottom panels display conidiophore length (µm) and width (µm). Different letters indicate significant differences among groups (ANOVA and Tukey’s HSD, p < 0.05). Whiskers indicate values within 1.5 times the interquartile range (IQR). Raw data is provided in Suppl. material 1: table S6.

Conidial dimensions (Fig. 1H) ranged from the smallest values in isolates from *F. rothmaleri* hosts belonging to Cab6x and Mon4x to the highest values in isolates from tetra- and octoploid *F. rothmaleri* hosts from the mixed population Can (Can4x, Can8x), with differences between the mean values of the two groups being marginally significant (Fig. 3; Suppl. material 1: table S6). Variability in conidial dimensions was mainly caused by differences in area (6.8 ± 0.9 *vs.* 7.3 ± 1.0 µm^2^) and length (4.3 ± 0.4 *vs.* 4.5–4.6 ± 0.4 µm); while width remained stable across populations (2.0–2.1 ± 0.2 µm). These values were not positively correlated in all cases with conidiophore length, which ranged from the shortest in endophytes isolated from Cab6x host plants (11.7 ± 0.2 µm) to the longest in those belonging to the tetraploid Mon4x population (13.1 ± 2.2 µm). Conidiophore width remained constant across all populations and cytotypes (1.8–1.9 ± 0.3–0.4 µm). The smallest values for conidia and conidiophore dimensions were found in endophytes isolated from the hexaploid population Cab6x (Fig. 3; Suppl. material 1: table S6). PERMANOVA tests revealed that all factors tested (individuals, source population, host ploidy level, and the interaction between source population and host ploidy level) contributed significantly to the variation in conidial morphology, being individual identity the factor that explained the largest proportion of variance (R² = 0.150, F_15_ = 5.331, p < 0.001; Suppl. material 1: table S7). The traits that most contributed to morphological differences at the group level (source population, host ploidy level, and the combination of both) were conidial length, width, and area and conidiophore length, which were significantly different in most pairwise comparisons, whereas conidiophore width remained consistently non-significantly different (Suppl. material 1: table S8). As a result, conidiophore width was excluded from the PCA due to its low contribution to group-level variance but was retained in the LDA models for its potential classification value.

**Figure 4.**
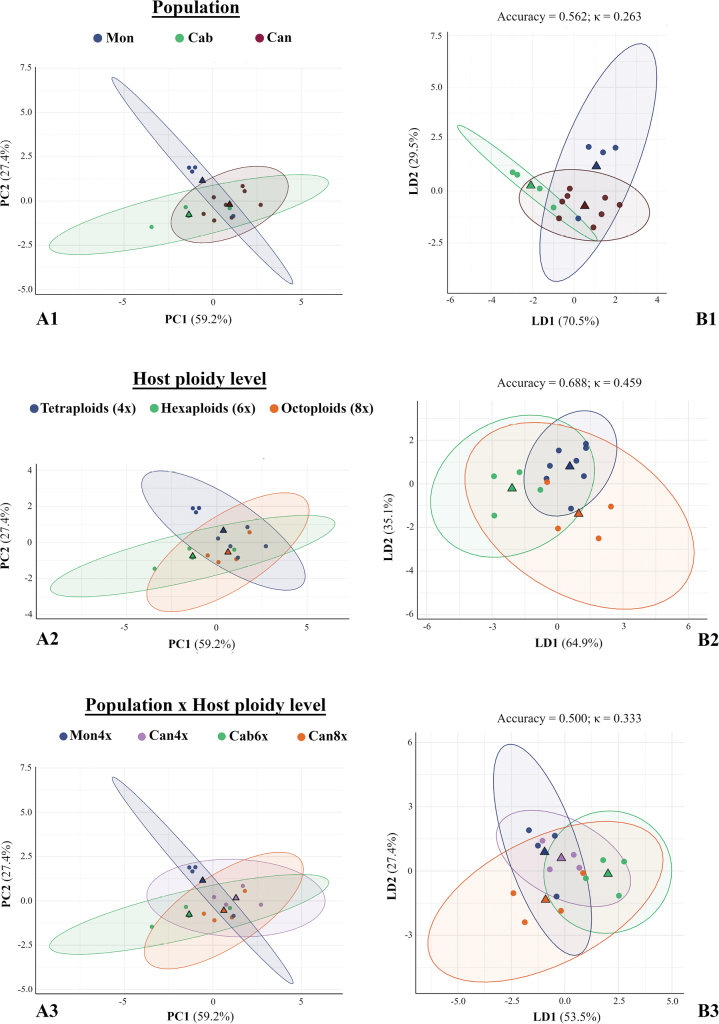
Bidimensional plots of multivariate morphological analyses of *Epichloë festucae* (n = 16, individual means). A. Principal Component Analysis (PCA) and B. Linear Discriminant Analysis (LDA), shown by grouping factors: source population (A1, B1), host ploidy (A2, B2), and their interaction (A3, B3). Axes indicate variance explained (PCA; Suppl. material 1: table S9) or trace proportion (LDA). LDA panels also show cross-validation accuracy and κ values (LOOCV; see Suppl. material 1: table S10). Triangles mark group centroids.

Principal component analyses (PCAs) revealed that morphological differen­tiation among *Epichloë* isolates was more clearly resolved at individual-level (n = 16; Fig. 4) and less at the replicate-level (n = 48; Suppl. material 1: fig. S3). When considering the resampling-level dataset (n = 469), no patterns were detected and consequently this dataset was not included in the results section. The cumulative variance explained by the first two principal components (PC1 and PC2) at individual-level was 86.6% (Suppl. material 1: table S9A); conidial traits (length, width and area) strongly loaded on PC1 (0.777), while conidiophore length dominated PC2 (0.936). PCA plots showed partially overlapping but distinguishable clusters by source population (Fig. 4) and ANOVA tests confirmed a significant effect of source population on PC1 and PC2 scores (p < 0.05), whereas ploidy had no consistent effect (Suppl. material 1: table S9B). Conversely, LDA models (Fig. 4, Suppl. material 1: fig. S3) using host ploidy level as the grouping factor achieved the highest cross-validated accuracy value, reaching up to at the individual level (68.8%; κ = 0.459; Fig. 4; Suppl. material 1: table S10). In all cases, classification models based on the interaction between population of origin and host ploidy level showed the weakest discriminative power (Suppl. material 1: table S10). Although these findings support source population as the main driver of morphological variation and host ploidy level as the most reliable predictor, variance proves to be very high within groups (see ellipses in Fig. 4, Suppl. material 1: fig. S3) despite the apparent patterns that emerge when considering the mean values in both datasets.

#### ﻿Genome size estimations

DAPI staining of asexual structures detected only one nucleus in all the *Epichloë festucae* isolates evaluated (Suppl. material 1: fig. S4), corroborating the suitability of our specimens for the flow cytometry approach. Genome size measurements of *E. festucae* isolates were reliable only within the first 6–10 days of culture; beyond this period, measurement quality declined markedly, with coefficients of variation (CV) exceeding 10%. Our genome size estimates showed mean genome size values of 0.044 ± 0.002 pg/1C for Mon4x samples, 0.045 ± 0.002 pg/1C for Can4x, 0.045 ± 0.003 pg/1C for Cab6x, and 0.046 ± 0.002 pg/1C for Can8x (Fig. 2; Suppl. material 1: table S11). No significant differences in genome size were detected between or within populations or host ploidy levels, evidencing the same ploidy level in all *E. festucae* samples from *F. rothmaleri* hosts, regardless of their cytotype (Figs 1I, 2).

#### ﻿Genetic characteristics and phylogenetic reconstruction

Single-gene alignments of the five loci considered were obtained for 33 accessions, including 8 newly sequenced *Epichloë* isolates from *Festuca rothmaleri* (Suppl. material 1: table S12), as well as 23 haploid *Epichloë* specimens and 2 *Claviceps purpurea* outgroups (Suppl. material 1: table S3). All sequences from these individuals passed our tests to assess the number of gene copies present in the genomes (orthologous single copy for protein-coding genes and one predominant ribotype for the ITS region). For the *tefA* gene, two different fragments were recovered and uploaded to the database independently. The newly obtained sequences from two *E. festucae* isolates from separate host plants of each *F. rothmaleri* population and ploidy level (Mon4x, Can4x, Cab6x and Can8x) were deposited in GeneBank as 48 new accessions (Suppl. material 1: table S12).

The multiple alignment of the *actG* dataset included 265 variable and potentially informative positions out of 1036 (25.6%), *CalM* 385 out of 1037 (37.1%), ITS 77 out of 517 (14.9%), *tefA* 202 out of 644 considering both recovered fragments (31.4%), and *tubB* 223 out of 1003 (22.2%). All maximum likelihood phylogenetic trees reconstructed separately from each alignment showed similar topologies and were consistent in placing the newly sequenced *Epichloë* specimens of *F. rothmaleri* within a strongly supported *E. festucae* clade containing other *E. festucae* sequences (Suppl. material 1: fig. S5). While the single-gene phylogenetic trees *actG*, ITS, and *tefA* showed different topological placements for the novel *E. festucae* sequences, interspersing those from other samples within the *E. festucae* clade (Suppl. material 1: fig. S5A, C, D), the single-gene phylogenetic trees *CalM* and *tubB* united the novel *E. festucae* isolated from *F. rothmaleri* into a monophyletic clade (Suppl. material 1: fig. S5B, E). The *CalM* gene alignment showed two polymorphic mutations specific to the novel *E. festucae* sequences at positions 699 and 731, which separated the novel samples from the rest of the *E. festucae* specimens, contributing to the high support of their *E. festucae*–*F. rothmaleri* subclade (98% BS; Suppl. material 1: fig. S5B). In contrast, in the *tubB* alignment, the four polymorphic positions were not private, with the novel *E. festucae* sequences sharing positions with *E. festucae* AL9436 (889), *E. festucae* Fl1 (940), and *E. festucae* FG1 (706, 916), which was reflected in the moderately supported *E. festucae*–*F. rothmaleri* subclade (74% BS; Suppl. material 1: fig. S5E). The newly generated *E. festucaeact*, *G*, ITS, and *tefA* gene sequences were virtually identical to those previously published (*E. festucae* AL9436, *E. festucae* Fl1, *E. festucae* FG1), with only a few single-bp polymorphisms varying between samples. Therefore, *CalM* alignment positions 699 and 731 could be potential new barcodes for the *E. festucae* samples isolated from *F. rothmaleri*.

**Figure 5.**
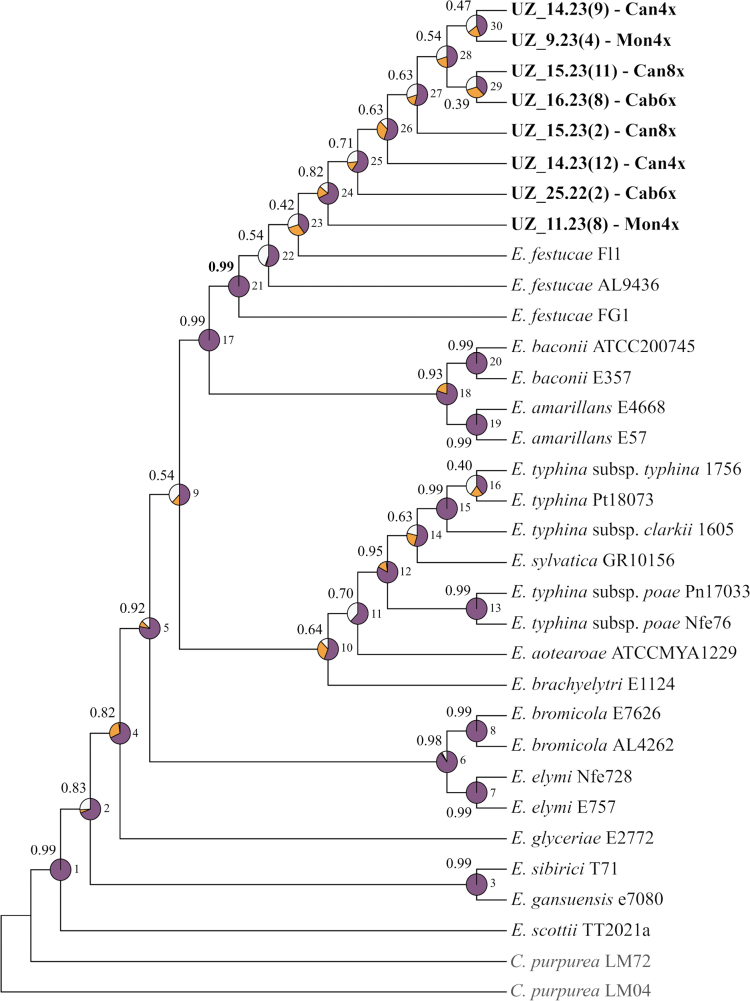
Multispecies coalescent tree of *Epichloë festucae* constructed with ASTRAL III using as inputs the independent *actG*, *CalM*, ITS, *tefA*, and *tubB* IQTREE2 ML trees (see Suppl. material 1: fig. S5). Newly analyzed samples (Suppl. material 1: table S12) are in bold; sequences of other *Epichloë* species and the outgroup *Claviceps purpurea* were obtained from NCBI (Suppl. material 1: table S3). Values on branches correspond to posterior probability support (PPS). Quartet values indicate nodal support for the best tree (q1; purple) and the first (q2; orange) and second (q3; white) alternative topologies. Node numbers are indicated in the phylogenetic tree and quartet values are listed in Suppl. material 1: table S13.

The multispecies coalescent (MSC) tree and the concatenated ML phylogenetic tree based on the dataset of the five *Epichloë* nuclear loci recovered topologies that were highly congruent with each other (Fig. 5, Suppl. material 1: fig. S6). In the MSC Astral tree, constructed from the five independent gene trees, nodes of the major *Epichloë* lineages showed strong overall posterior probability support (PP) values and high q1 values, supporting the best topology (Fig. 5; Suppl. material 1: table S13). Similarly, the concatenated ML tree showed high bootstrap support values and moderate to low SCFL values for the main *Epichloë* clades (Suppl. material 1: fig. S6). Both phylogenies retrieved a strongly supported *E. festucae* clade (0.99 PP and q1 = 1 in the Astral species tree, 100 BS and 73.3% SCFL in the concatenated ML tree). Within this clade, the new *E. festucae* samples from *F. rothmaleri* coalesced into an independent lineage showing high (0.82 PP, q1 = 0.67, in the Astral tree) to moderate (53% BS, 37.6% SCFL, in the concatenated ML tree) support.

## ﻿Discussion

### ﻿The polyploid endemic species *Festuca rothmaleri* hosts a haploid *Epichloë festucae* strain

This study demonstrates the existence of a geographic area where *F. rothmaleri* individuals of three known ploidy levels (Figs 1F, G, 2, Suppl. material 1: fig. S1; Suppl. material 1: table S4) could live in sympatry or, at least, in probable occasional contact with each other due to the outcrossing nature, wind-mediated pollination, and seed dispersal zoochory of this grass (Catalán 2006). As in other *Festuca* taxa (x = 7; Catalán 2006; Martínez-Sagarra et al. 2021), these cytotypes show genome sizes of 7.54 ± 0.21 pg/2C (4x), 12.81 ± 0.16 pg/2C (6x), and 15.58 ± 0.28 pg/2C (8x), supported by chromosome counts (Fig. 1F). These values match other members of F. sect. Aulaxyper (Šmarda et al. 2008; Moreno-Aguilar et al. 2022) except for tetraploids, and unlike many high-polyploids in the group, they do not show the strong monoploid genome reduction (1Cx) reported elsewhere (Šmarda et al. 2008; Garnatje et al. 2023).

Incidence rates of *Epichloë* endophytes in *Festuca* species can vary greatly depending on the population and the holobiont considered, ranging from 10% to almost 100% (e.g., Siegel et al. 1984; Zabalgogeazcoa et al. 2006b; Wäli et al. 2007; Iannone et al. 2009; Gundel et al. 2014; Mcgranahan et al. 2015). The establishment of this symbiotum has proven to be advantageous for the host as long as the endophyte is asexually transmitted, without forming stromata (Xia et al. 2018; Wang et al. 2020; Hewitt et al. 2021). Thus, for mainly asexual species such as *E. festucae*, the enhanced tolerance that this interaction promotes against abiotic stress factors may positively select the high incidence of this endophyte in *F. rothmaleri* populations in the NW of the Iberian Peninsula, which is known to have dry periods and harsh conditions during the flowering season of this grass. Supporting this hypothesis, the highest incidence rate (90.9%) was detected in a dehesa ecosystem (Cab), which is characterized by high coverage of herbaceous pasture (Prieto-Guijarro et al. 1999). In contrast, the other two sampling spots are located in forested and humid areas, close to streams (Mon, Can). Interestingly, the host’s ploidy level does not appear to limit the endophytic association, since the host polyploids (4x, 6x and 8x) did not display wide variation in the incidence rates, pointing out the high adaptability of this *E. festucae* strain to the diverse physiological contexts.

Exploratory mating-type screening showed that the *Epichloë festucae* strain infecting *Festuca rothmaleri* harbors both mating types across all source populations, yet no stromata were observed, suggesting that sexual reproduction is either absent or extremely rare. Stromata are essential for horizontal transmission and recombination in *Epichloë* (Leuchtmann et al. 2014), and vertical inheritance through seed would maintain a single maternal genotype. Intraspecific ploidy variation has not been reported in *Epichloë*, and heteroploids appear to arise mainly through interspecific hybridization, often involving *E. typhina* (Tsai et al. 1994; Leuchtmann et al. 2014). For such events, ascospores from stromata of another species would need to infect ovules already colonized by *E. festucae*. Although stromata of *E. typhina* are common on sympatric grasses (e.g., *Dactylis*, *Holcus*, *Brachypodium*, *Lolium*; personal observations), hybridization with *F. rothmaleri* seems rare, likely due to host specificity and biochemical barriers. Occasional exceptions (e.g., *L. perenne*; Moon et al. 2004) show that barriers can fail, but our findings suggest that this endophyte might persist as a haploid vertically transmitted lineage. That said, recent research has shown that certain *Epichloë* species can produce asexual conidia on the surface of leaf blades, and experimental evidence indicates that these epiphytic spores may facilitate water-mediated horizontal dispersal (Tadych et al. 2007; Tadych et al. 2014; Becker et al. 2016). It would therefore be valuable to examine whether epiphytic conidia are produced in this host–endophyte system.

Morphological analyses assessing the asexual reproductive structures revealed phenotypic plasticity in *Epichloë festucae* (Leuchtmann and Schardl 2005). In our study, both source population and host ploidy level influenced morphological traits, with conidial size showing the clearest differentiation among groups (Fig. 4, Suppl. material 1: fig. S3; Suppl. material 1: tables S6–S10). Host ploidy was a stable predictor across models, suggesting a possible biological effect. All *Festuca rothmaleri* cytotypes appear to host the same *E. festucae* strain, producing asexual spores measuring 4.4 ± 0.4 [3.4–5.5] µm × 2.0 ± 0.2 [1.5–2.6] µm and conidiophores 12.4 ± 2.3 [5.7–18.7] µm × 1.8 ± 0.4 [1.0–2.9] µm (Fig. 3; Suppl. material 1: table S6). We also report the first estimate of conidial area (7.1 ± 1.0 [4.8–9.8] µm^2^), a potentially informative trait given the diversity of spore shapes in *Epichloë* (Iannone et al. 2009; McCargo et al. 2014; Thünen et al. 2022). These dimensions overlap with haploid strains such as *E. festucae* E189 and *E. typhina* E8 (Leuchtmann 1994; Kuldau et al. 1999) but are smaller than those of heteroploid taxa like *E. coenophiala* E19 (8.2 ± 1.4 × 2.2 ± 0.5 µm) and *E. hybrida* Lp1 (5.5 ± 0.5 × 2.4 ± 0.3 µm). Overall, spore shape and size are consistent with previously described haploid *E. festucae* isolates (Zabalgogeazcoa et al. 1999; Gundel et al. 2014). Regarding the macroscopic characteristics of the *E. festucae* cultures, the growth rates of all specimens were similar and did not present significant differences considering any of the grouping factors (Suppl. material 1: fig. S2). This result demonstrates that this *E. festucae* strain maintains a consistent growth rate regardless of its origin and can be used as a defining characteristic when considering the same experimental conditions. Furthermore, high phenotypic plasticity was detected in terms of appearance, which is consistent with previous studies that have reported this characteristic in this genus (Du et al. 2024).

Genome size has been estimated for only a handful of *Epichloë* taxa, traditionally by electrophoretic karyotyping or quantitative Southern blotting (Kuldau et al. 1999) and more recently through genome sequencing (e.g., Winter et al. 2018; Thünen et al. 2022). Reported haploid genomes range from ~26 Mb in *E. elymi* E757 to ~45 Mb in E. typhina subsp. clarkii 1605, with *E. festucae* strains typically spanning ~29–36 Mb. Heteroploid species such as *E. uncinata* e167 (diheteroploid; 53 Mb) and *E. coenophiala* 1033/212 (triheteroploid; 84–88 Mb; Lee et al. 2025) are substantially larger. Some discrepancies exist between methods; for example, *E. typhina* E8 was estimated at 28.8 Mb by karyotyping but 41.4 Mb by sequencing, likely reflecting technical biases and the structural complexity characteristic of *Epichloë* genomes (Treindl et al. 2021). Here we provide the first flow cytometry estimates for *Epichloë*. All *E. festucae* isolates from *F. rothmaleri* contained a single nucleus and averaged 0.045 ± 0.002 pg/1C (~42.8 Mb), slightly larger than many published *E. festucae* assemblies but still within the haploid range and close to *E. typhina*. These results underscore lineage-specific variation and suggest that genome size within *Epichloë* may vary more than previously recognized. Importantly, flow cytometry offers a rapid, scalable way to capture this diversity. Expanding this approach across the genus could clarify ploidy, reveal structural patterns, and improve our understanding of genome evolution in these ecologically and agronomically important symbionts.

Lastly, the phylogenetic analyses of five nuclear loci are highly consistent in placing our *Epichloë* specimens within the *E. festucae* clade (Fig. 5, Suppl. material 1: figs S5, S6). All five loci showed strong phylogenetic signal since, as the overall topology recovered for the species is very similar to that obtained by Thünen et al. (2022) using 2828 single-copy genes. Therefore, although some supporting values (quartet frequencies and SCFL) should be interpreted with caution due to the low number of gene trees considered, our phylogenies proved to be reliable, and the use of these five nuclear loci allowed us to unambiguously classify our samples within a separate monophyletic lineage of the *E. festucae* clade. Further whole-genome analyses will be essential to refine the phylogenetic placement of these isolates and to uncover potential adaptive or genomic features unique to this lineage.

Altogether, these results provide the first detailed view of this host–endo­phyte association and establish a robust baseline for future functional and evolutionary studies.

### ﻿A unique framework: asymmetric ploidy in the *Festuca*–*Epichloë* symbiosis

Polyploidy is a major driver of plant evolution, altering physiology, morphology, and ecological performance, and often reshaping interactions with other organisms (Chen 2007; Heslop-Harrison et al. 2023). In grasses, polyploidy is linked to increased adaptability, changes in symbiont incidence, and shifts in community dynamics (Dirihan et al. 2016; Saether 2024). At the genomic scale, whole-genome duplication affects gene expression, epigenetic regulation, and chromosome architecture, all of which may influence compatibility with symbionts (Bonfante and Genre 2010; Madlung 2013). Fungal endophytes of the genus *Epichloë* reflect these dynamics: several taxa are natural heteroploids formed through interspecific hybridization and carry larger, more complex genomes (*E. coenophiala*, *E. uncinata*, *E. tembladerae*, *E. novae-zelandiae*; Moon et al. 2004; Chen et al. 2009; Leuchtmann et al. 2019; Lee et al. 2025). These hybrid, asexual endophytes often occur in hybrid or polyploid hosts, as in the classic *Festuca arundinacea*–*E. coenophiala* system, where host allopolyploidization paralleled endophyte hybridization (Schardl et al. 1994, 2004; Suppl. material 1: table S1).

In contrast, *Festuca rothmaleri* presents an unusual scenario: across its three cytotypes (tetraploid, hexaploid, octoploid), all plants host the same haploid *E. festucae* strain, confirmed by identical barcodes and congruent phylogenies (Figs 2, 5; Suppl. material 1: tables S4, S11). This asymmetry—a polyploid host paired with a haploid symbiont—has not been documented before in the genus and, to our knowledge, represents the first detailed report of a haploid *Epichloë* consistently infecting multiple polyploid host cytotypes. Although the auto- or allopolyploid origins of these cytotypes remain unclear, the consistent association suggests strong host specificity and long-term compatibility. Crucially, it raises new questions about how a single fungal genotype copes with the genomic and physiological changes imposed by host polyploidy. Do different cytotypes present differences in the production and diversity of protective alkaloids? Could polyploid hosts alter endophyte gene expression or metabolic pathways? Do these holobionts, despite sharing the same haploid *Epichloë*, differ in their responses to drought, pathogens, or grazing? Might cytotype diversity impact endophyte population structure and genetic stability over time, with downstream effects on host fitness, plant community composition, and ecosystem processes? These questions highlight a particularly important area for future research in which this system provides a rare opportunity to isolate the effects of plant genome duplication on a stable fungal partner. Coupled with the methodological advances introduced here—digital morphometric analysis of conidia and the first flow-cytometry genome size estimates for *Epichloë*—the ploidy-asymmetric symbiosis between *F. rothmaleri* and *E. festucae* offers a powerful model to examine how genomic complexity and symbiosis co-evolve, with implications for basic research and grassland management.

## ﻿Conclusions

In this study, by integrating cytogenetic, morphological, and molecular approaches, and introducing two methodological advances—flow cytometry for *Epichloë* genome size estimation and digital conidial morphometry—a previously undocumented symbiosis is described: a haploid *Epichloë festucae* strain consistently colonizing the Iberian endemic *Festuca rothmaleri* across its three polyploidy cytotypes (4x, 6x, 8x). This stability against a backdrop of host polyploidy contrasts with other *Loliinae*–*Epichloë* associations, where both partners often show increased genomic complexity. We establish a solid baseline for future work, providing a useful framework to explore how host genome variation influences symbiotic partners and provides tools that can be applied to other grass–endophyte interactions.

## ﻿Additional information

### Use of AI

No use of AI was reported.

### Adherence to national and international regulations

All the fungal strains used in this study have been legally obtained, respecting the Convention on Biological Diversity (Rio Convention).

### Author ORCIDs

Alba Sotomayor-Alge https://orcid.org/0000-0003-2317-0873

Luis A. Inda https://orcid.org/0000-0002-1214-375X

Ernesto Ángel-Beamonte https://orcid.org/0009-0002-6714-7106

Íñigo Zabalgogeazcoa https://orcid.org/0000-0002-9524-7799

Pilar Catalán https://orcid.org/0000-0001-7793-5259

### Data availability

The sequence data generated for this project is available in the NCBI GeneBank database under the accession numbers summarized in Suppl. material 1: table S12. The data and scripts underpinning the analysis reported in this paper are deposited in the associated Dryad (https://doi.org/10.5061/dryad.hx3ffbgsb) and GitHub (https://github.com/Bioflora/FrothmaleriEpichloe) repositories. Georeferenced sampling records have been deposited in GBIF.

## Supplementary materials

Supplementary material 1 Suppl. appendices S1, S2, figs S1–S6, tables S1–S13 Data type docx Explanation note **figure S1.** Geographic map showing the locations of the *Festuca rothmaleri* populations containing *Epichloë festucae* endophytes under study in NW Spain (Salamanca province). **figure S2.** Growth of *Epichloë festucae* cultures on 5.5 cm PDA plates at 2 days (A1–D1), 1 week (A2–D2), 2 weeks (A3–D3), and 3 weeks (A4–D4) after inoculation. **figure S3.** Bidimensional plots of multivariate morphological analyses of *Epichloë festucae* (n = 48, replicate means). **figure S4.** Asexual structures of *Epichloë festucae* stained with DAPI to visualize nuclei. **figure S5.** Maximum-likelihood phylogenetic trees (IQ-TREE2) of *Epichloë festucae* based on five nuclear loci: (A) γ-actin (*actG*), (B) calmodulin (*CalM*), (C) ITS region, (D) translation elongation factor 1-α (*tefA*), and (E) β-tubulin (*tubB*). **figure S6.** Concatenated maximum-likelihood phylogeny of *Epichloë festucae* based on five nuclear loci (*actG*, *CalM*, ITS, *tefA*, and *tubB*), constructed with IQ-TREE2. **table S1.** Ploidy information for 22 pooid host grasses and their *Epichloë* endophytes. **table S2.** Information on primers pairs and PCR programs used in the amplification of the five barcoding loci (*actG*, *CalM*, ITS, *tefA*, *tubB*) and both mating type idiomorphs (MAT1-1 and MAT1-2) studied in *Epichloë festucae* samples. **table S3.** Reference genomes of *Epichloë* spp. and the outgroup (*Claviceps purpurea*) species, obtained from NCBI, whose barcoding sequences of the 5 loci were included in the phylogenetic analysis of the *Epichloë festucae* samples under study, isolated from their *Festuca rothmaleri* host plants. **table S4.** Genome size estimations (pg/2C) and chromosome counts for the studied samples of *Festuca rothmaleri* at individual and population levels. The study included tetraploid [Mon4x (A), Can4x (B)], hexaploid [Cab6x (C)] and octoploid [Can8x (D)] individuals. **table S5.** PCR-based exploratory screening of mating types MAT1 (785 bp) and MAT2 (215 bp) in *Epichloë* isolates from the three sampling populations (Mon, Cab, Can), using primers listed in Suppl. material 1: table S2. **table S6**. Morphological analysis of asexual characters (conidia, conidiophores) of *Epichloë festucae* isolates from host plants of each of the four *Festuca rothmaleri* populations and host ploidy levels under study (labelled here as “Pop”; Mon4x, Can4x, Cab6x, Can8x). **table S7.** Summary of PERMANOVA and variance homogeneity (betadisper) tests for each grouping factor (individual, population of origin, host ploidy level, and the interaction between population and host ploidy level) of the morphological characters of *Epichloë festucae* studied. **table S8.** Results of pairwise permutation tests (10,000 resamples) assessing differences in morphological traits of the *Epichloë festucae* samples studied considering three grouping factors. **table S9.** Principal Component Analyses and ANOVAs of morphological traits of *Epichloë festucae* studied across data aggregation levels. **table S10.** Performance and discriminant coefficients of Linear Discriminant Analysis (LDA) of morphological traits of *Epichloë festucae* studied by dataset and grouping factor. **table S11.** Genome size estimates (pg/1C) measured through flow cytometry analysis in *Epichloë festucae* endophytes isolated from host *Festuca rothmaleri* individuals from each population and ploidy group under study (Mon4x, Can4x, Cab6x, Can8x). **table S12.** NCBI GeneBank accession codes for the five nuclear loci [*actG*, *CalM*, ITS, *tefA* (frag1 and frag2), *tubB*] sequenced from *Epichloë festucae* specimens under study isolated from their *Festuca rothmaleri* host plants. **table S13**. Nodal quartet support values for the multispecies coalescent tree of *Epichloë festucae*. **appendix S1.***Epichloë* Conidia: A software for morphometric analysis of asexual fungal structures. **appendix S2.** Detailed modified CTAB protocol for *Epichloë* DNA extraction. https://binary.pensoft.net/file/1448098This dataset is made available under the Open Database License (http://opendatacommons.org/licenses/odbl/1.0/). The Open Database License (ODbL) is a license agreement intended to allow users to freely share, modify, and use this Dataset while maintaining this same freedom for others, provided that the original source and author(s) are credited.Alba Sotomayor-Alge, Luis A. Inda, Ernesto Ángel-Beamonte, Íñigo Zabalgogeazcoa, Pilar Catalán
