# Supplementary material for: ﻿Unveiling an asymmetric plant–fungal symbiosis: morphological, cytogenetic, and molecular characterization of a haploid Epichloë festucae strain associated with three polyploid cytotypes of the Iberian endemic grass Festuca rothmaleri
Source: IMA Fungus. 2025 Oct 24;16:e162692. doi: 10.3897/imafungus.16.162692 (PMC12579330; doi:10.3897/imafungus.16.162692)
Supplement: Supplementary material 1 — Suppl. appendices S1, S2, figs S1–S6, tables S1–S13 [file imafungus-16-e162692-s001.docx]

**Supplementary Material**

**Appendix S1.** *Epichloë* Conidia: A software for morphometric analysis of asexual fungal structures.

Image-based analysis in fungal biometrics enables accurate measurement of morphological features such as conidiophore segment length and conidial area, parameters that are often difficult to estimate directly. Obtaining reliable measurements requires high-quality micrographs captured with minimal geometric distortion and consistent imaging conditions (objective, magnification, aspect ratio, and camera settings) to ensure metric comparability and shape fidelity between image batches.

We developed *Epichloë* Conidia, a custom application for the analysis of JPG images captured at 1000× magnification using an optical microscope with an integrated digital camera. The images are 1920 × 1280 pixels in size, with a resolution of 15.8097 pixels/µm, calibrated to a micrometer standard. The software, developed in MATLAB R2017b, includes two main modules accessible from a central menu. The workflow begins with image import, followed by: (i) green channel equalization, (ii) edge enhancement, (iii) binary segmentation using a refined Otsu thresholding algorithm, and (iv) background noise removal based on empirically determined pixel thresholds.

Length measurements were made manually by selecting two points with the mouse; the number of white pixels between them was counted and converted to microns using the calibrated resolution mentioned above. Conidial area measurements were automated. After preprocessing, the software identifies connected components in the binary image, retains those that exceed a minimum size, and filters out elliptical shapes with an eccentricity < 0.95, typical of *Epichloë* conidia. The area (µm²) is calculated by dividing the number of pixels by the square of the resolution (15.8097²). The measurement results are displayed directly on the processed image for visual verification.

**Appendix S2.** Detailed modified CTAB protocol for *Epichloë* DNA extraction.

After the culture had grown for 7–10 days on potato dextrose agar (PDA; Potato Dextrose Agar EP/USP/BAM, Condalab) using a single sterile cellophane disc, the marginal (youngest) parts of the culture (approximately 1 cm²) were harvested and ground in two 20–30-second rounds using 30–40 zirconium microspheres (400 μm in diameter). Centrifugation pulses were interspersed during grinding to promote tissue disruption. The mycelium was then resuspended in 500 μl of prewarmed (65°C) 2x CTAB extraction buffer, and 1 μl of β-mercaptoethanol (2-mercaptoethanol ≥98%, SIGMA) was added. The samples were mixed vigorously and incubated for 1 hour at 65°C, with tube inversions every 10 minutes to facilitate DNA disruption and release. After cooling for 5 minutes at room temperature, the samples were centrifuged at maximum speed for 8 minutes. The supernatant was then transferred to a new sterile tube where 2 µl of RNase A/T1 (Thermo Scientific; 2 mg/ml) was added and incubated for 15 minutes at 37°C. An equal volume of phenol:chloroform:isoamyl alcohol (25:24:1) was then added and mixed by multiple inversions. The samples were then centrifuged at maximum speed (14,000 rpm) for 10 minutes, and the upper aqueous phase was transferred to another tube, where an equal volume of chloroform:isoamyl alcohol (24:1) was added and mixed before another centrifugation step under the same conditions. The upper aqueous phase was transferred again to a new tube, and this last step was repeated once more. Subsequently, 0.7 volumes of ice-cold isopropanol were added, and the samples were gently inverted every 2 minutes during a 10-minute incubation step at room temperature. The samples were subjected to a centrifugation step at maximum speed (14,000 rpm) for 5 minutes and the supernatant was carefully removed without disturbing the DNA pellet. The DNA pellet was washed twice with 500 μl of 70% ethanol for 15 minutes, followed by a final centrifugation step under the same conditions mentioned above. Finally, the ethanol was removed, and the pellet was air-dried for approximately 15 minutes before being resuspended in 30 μl of prewarmed ddH₂O (37 °C). To ensure complete resuspension of the DNA, it was left overnight at 4 °C before performing quality control checks and using it for subsequent analyses.

**
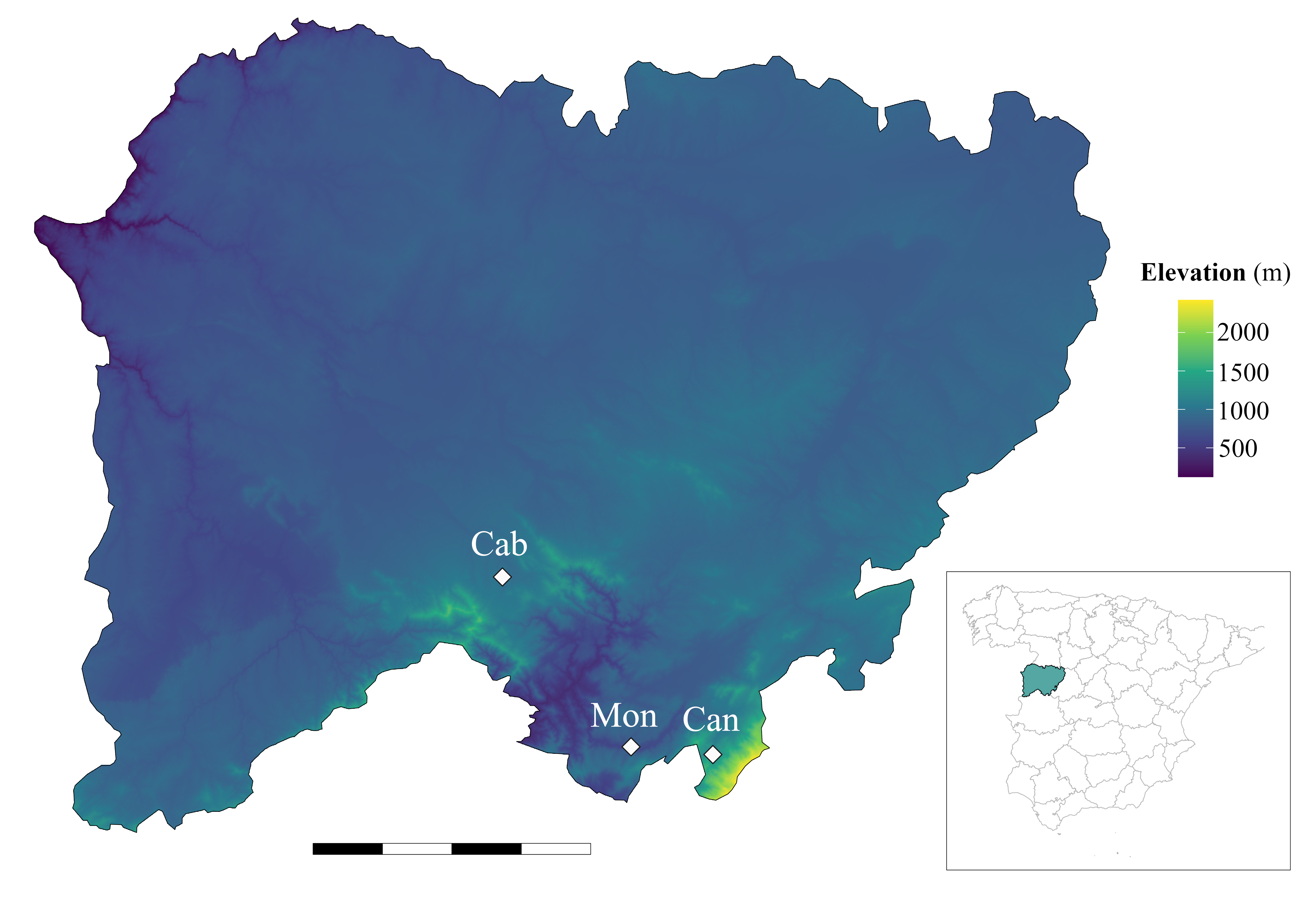
**

**Figure S1.** Geographic map showing the locations of the *Festuca rothmaleri* populations containing *Epichloë festucae* endophytes under study in NW Spain (Salamanca province). The three sampled populations Montemayor del Río (Mon; 40°20'49.7"N, 5°54'54.4"W), Candelario (Can; 40°20'17.5"N, 5°45'59.2"W), and El Cabaco (Cab; 40°34'02.6"N, 6°08'00.9"W) are separated by more than 10 km and mountain ranges, representing three different altitudes and ecosystems. Elevation ranges indicated in the figure; scale bar: 40 km. These georeferenced sampling records have been deposited in GBIF.


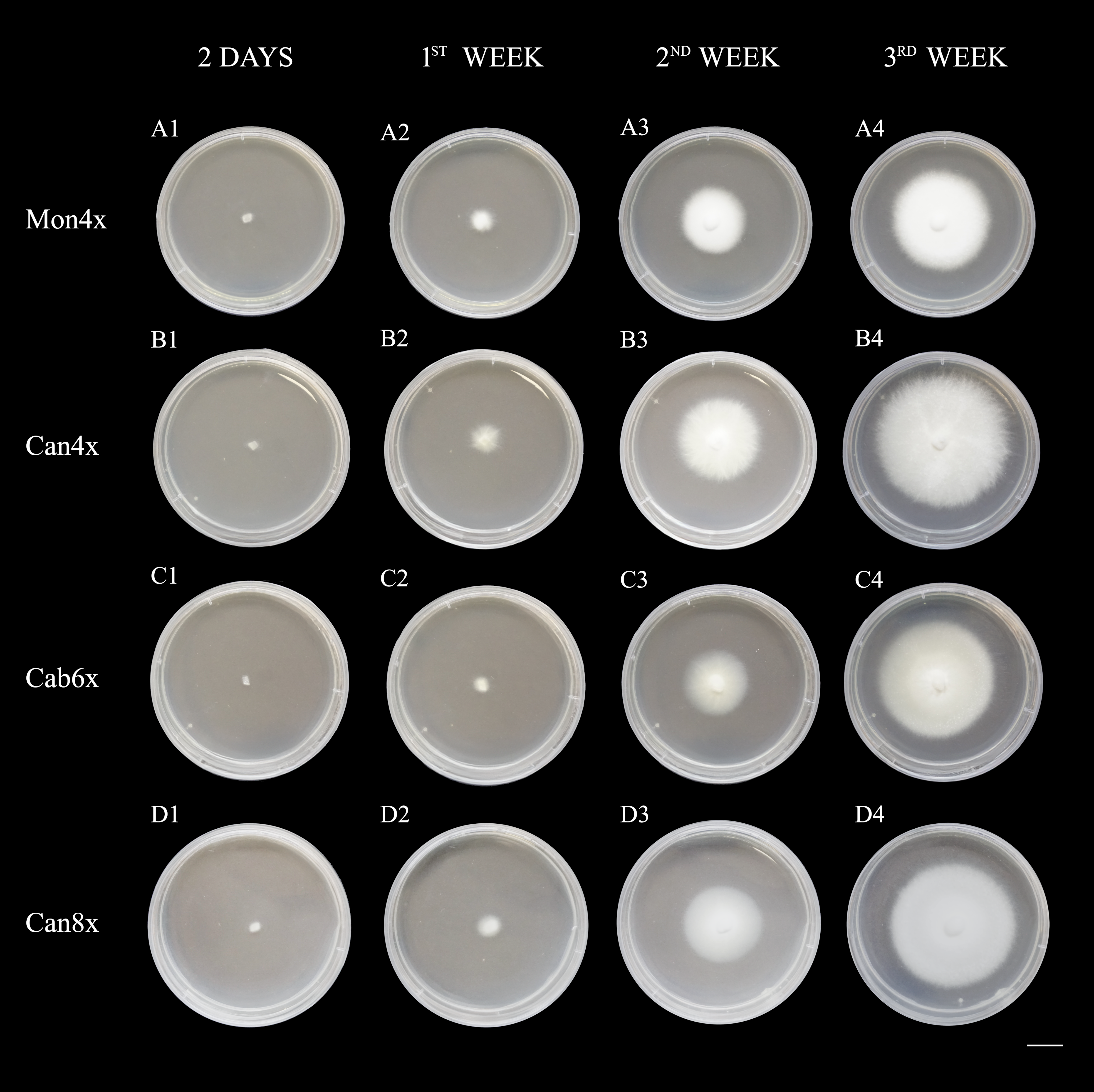


**Figure S2.** Growth of *Epichloë festucae* cultures on 5.5 cm PDA plates at 2 days (A1–D1), 1 week (A2–D2), 2 weeks (A3–D3), and 3 weeks (A4–D4) after inoculation. Rows A–D show different genotypes isolated from *Festuca rothmaleri* populations with varying ploidy: (A) UZ_10.23(4)-Mon4x, (B) UZ_14.23(9)-Can4x, (C) UZ_16.23(1)-Cab6x, (D) UZ_15.23(10)-Can8x. Scale bar: 1 cm.


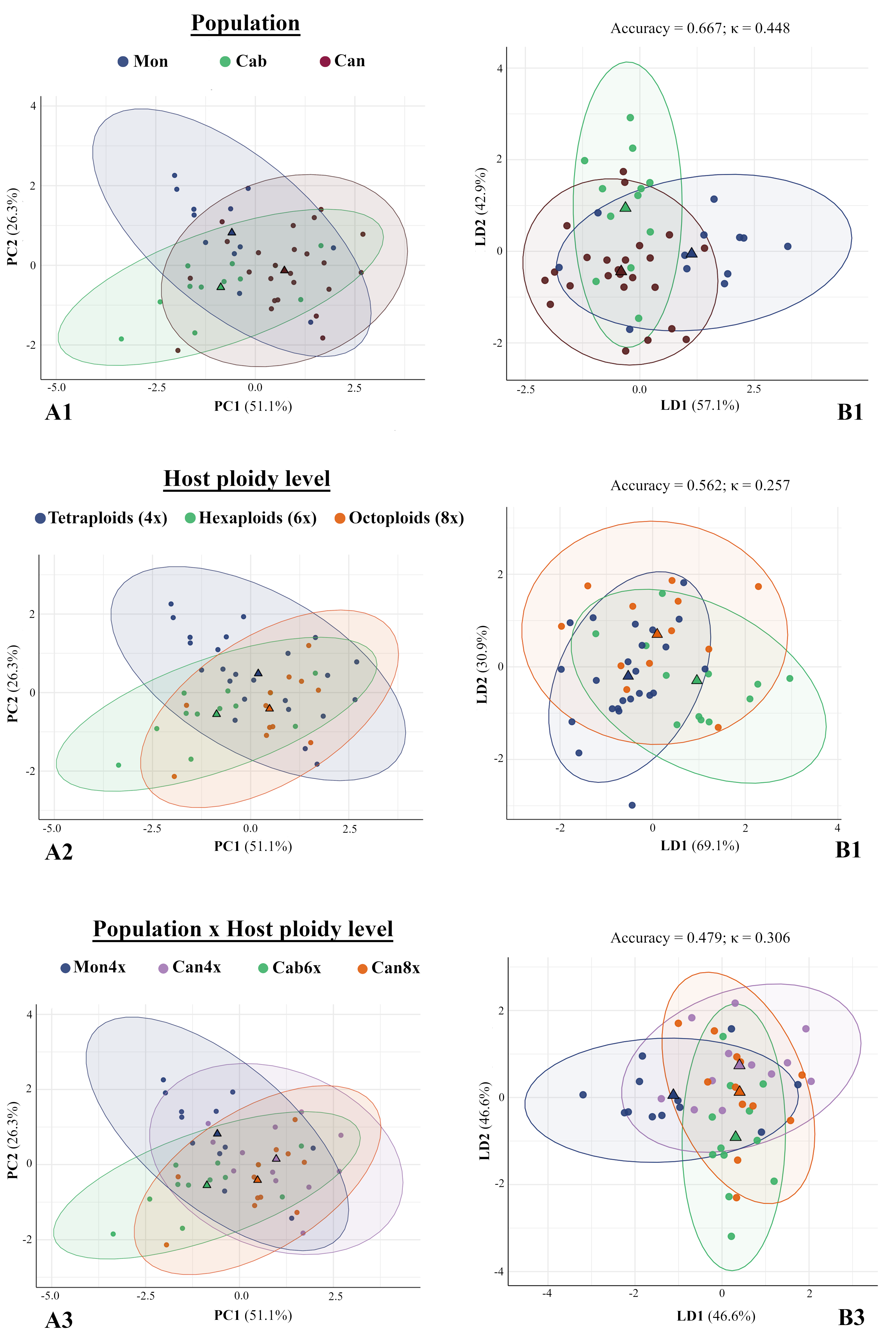


**Figure S3.** Bidimensional plots of multivariate morphological analyses of *Epichloë festucae* (n = 48, replicate means). (A) Principal Component Analysis (PCA) and (B) Linear Discriminant Analysis (LDA), shown by grouping factors: source population (A1, B1), host ploidy (A2, B2), and their interaction (A3, B3). Axes indicate variance explained (PCA; Table S9) or trace proportion (LDA). LDA panels also show cross-validation accuracy and κ values (LOOCV; see Table S10). Triangles mark group centroids.

**
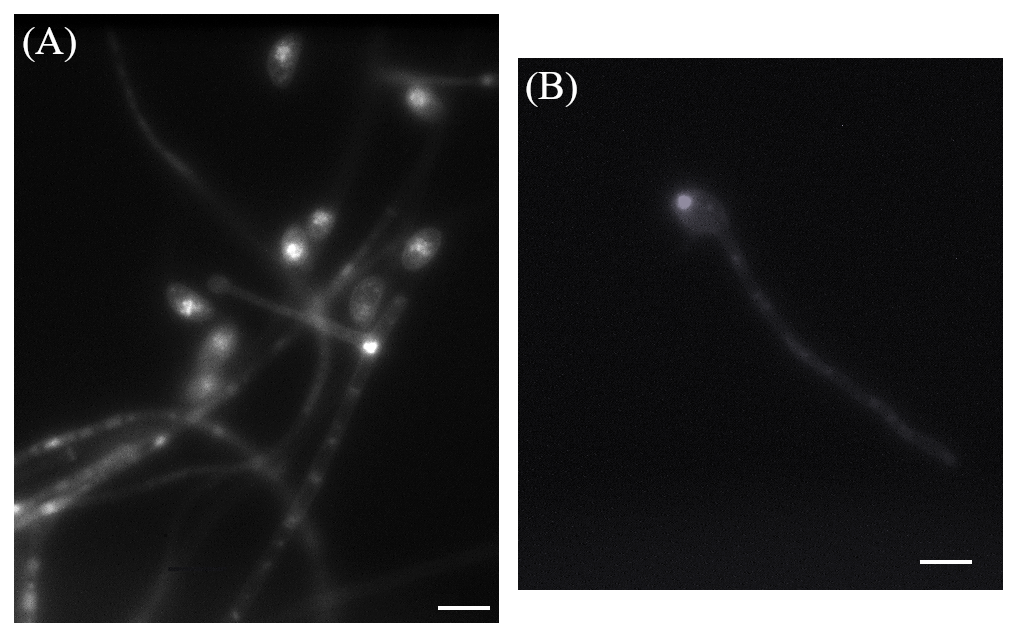
**

**Figure S4.** Asexual structures of *Epichloë festucae* stained with DAPI to visualize nuclei. (A) Conidia and mycelia containing a single nucleus. (B) Conidia germinating into new mycelia. Images at 100× magnification. Scale bars: 5 µm.

**
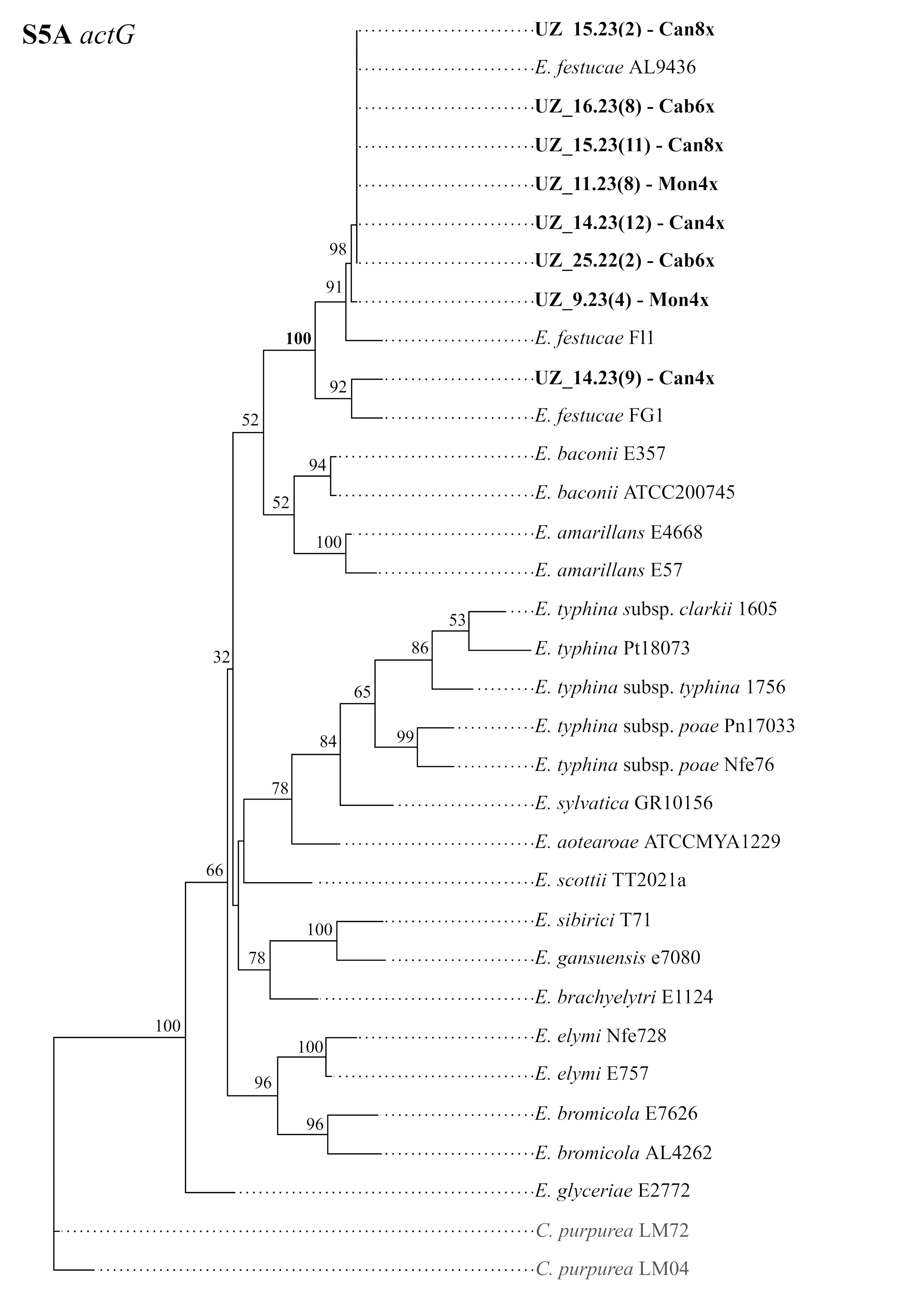
**

**Figure S5A.** Full figure legend provided with panel S5E.


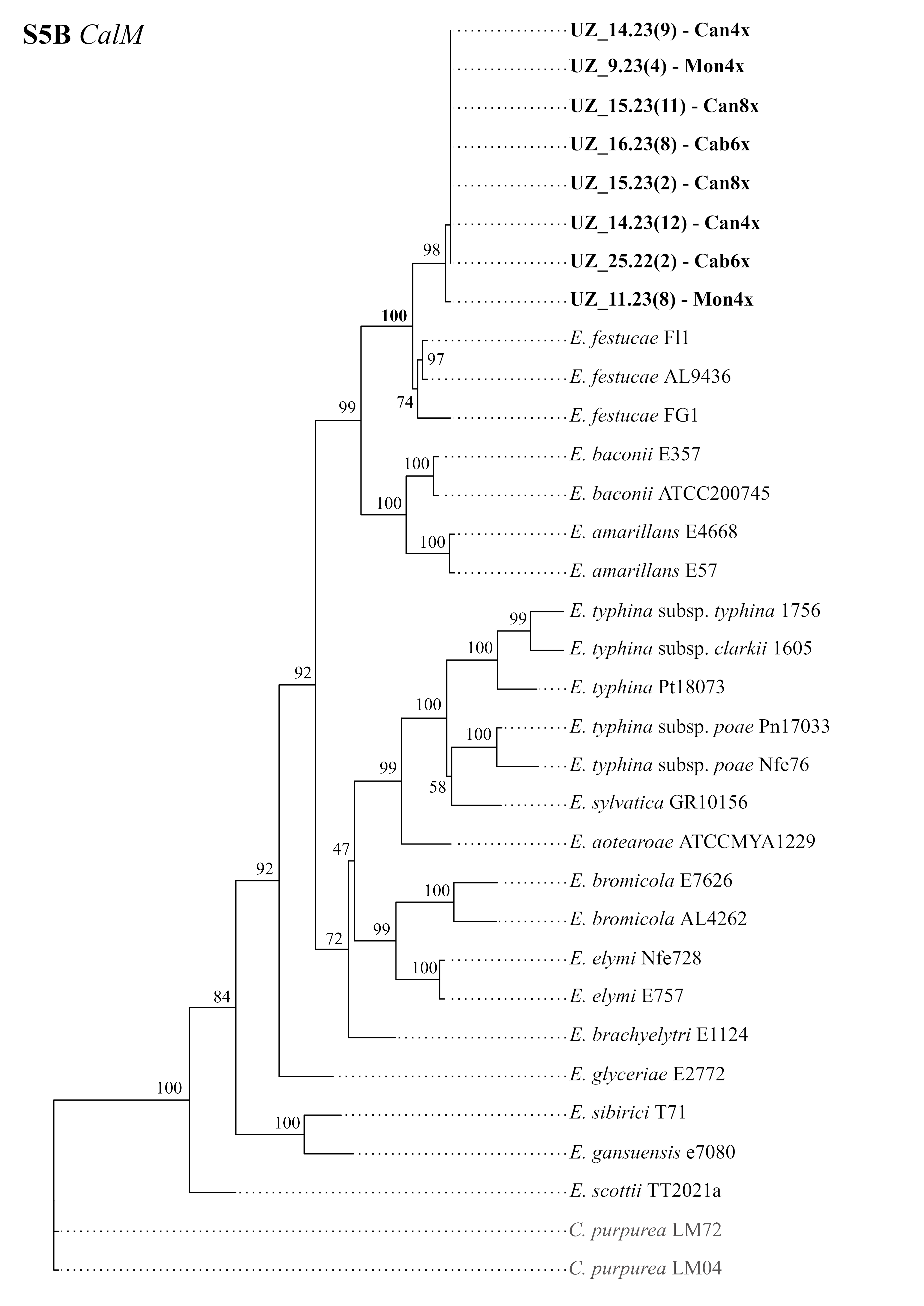


**Figure S5B.** Full figure legend provided with panel S5E.


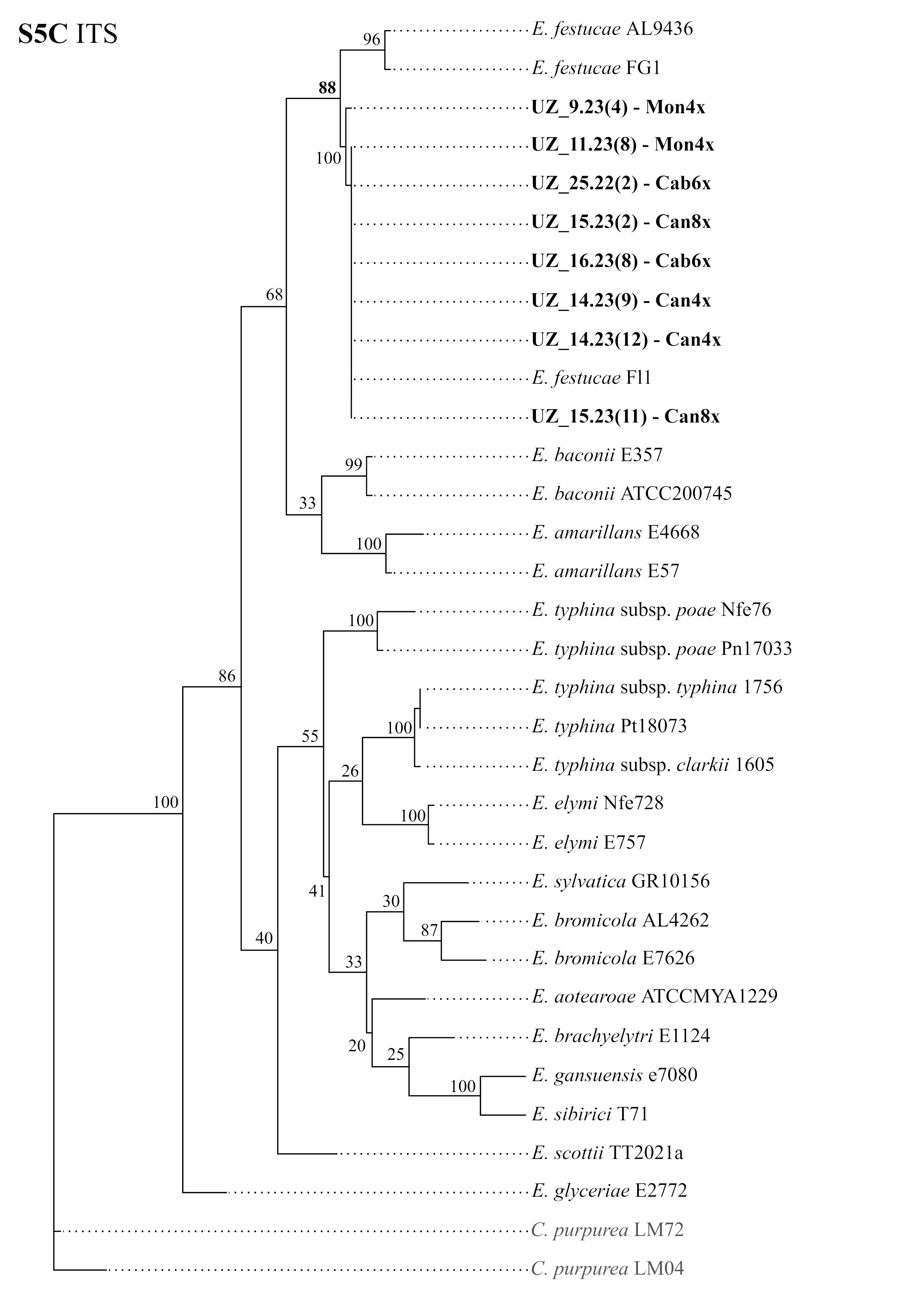


**Figure S5C.** Full figure legend provided with panel S5E.


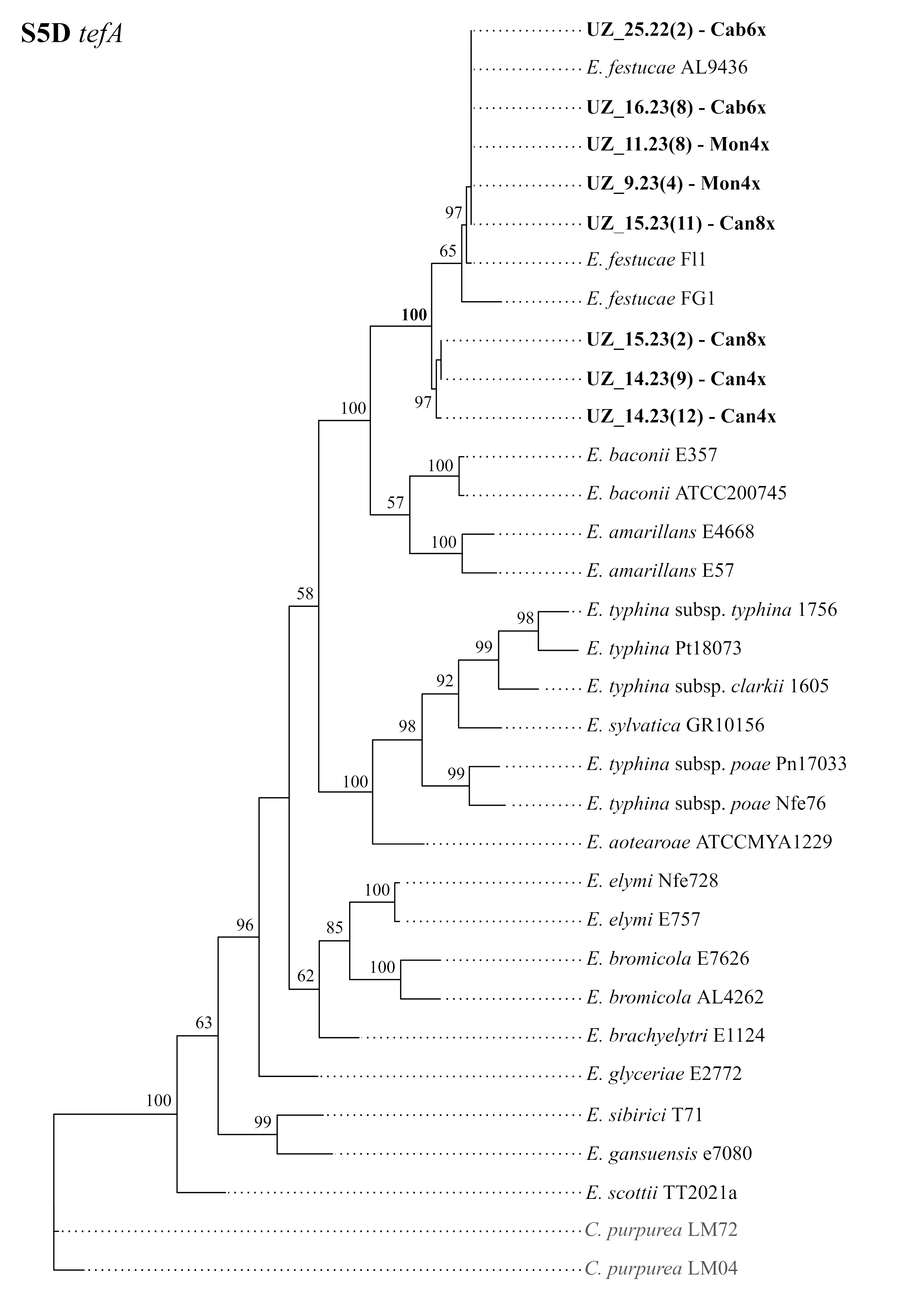


**Figure S5D.** Full figure legend provided with panel S5E.


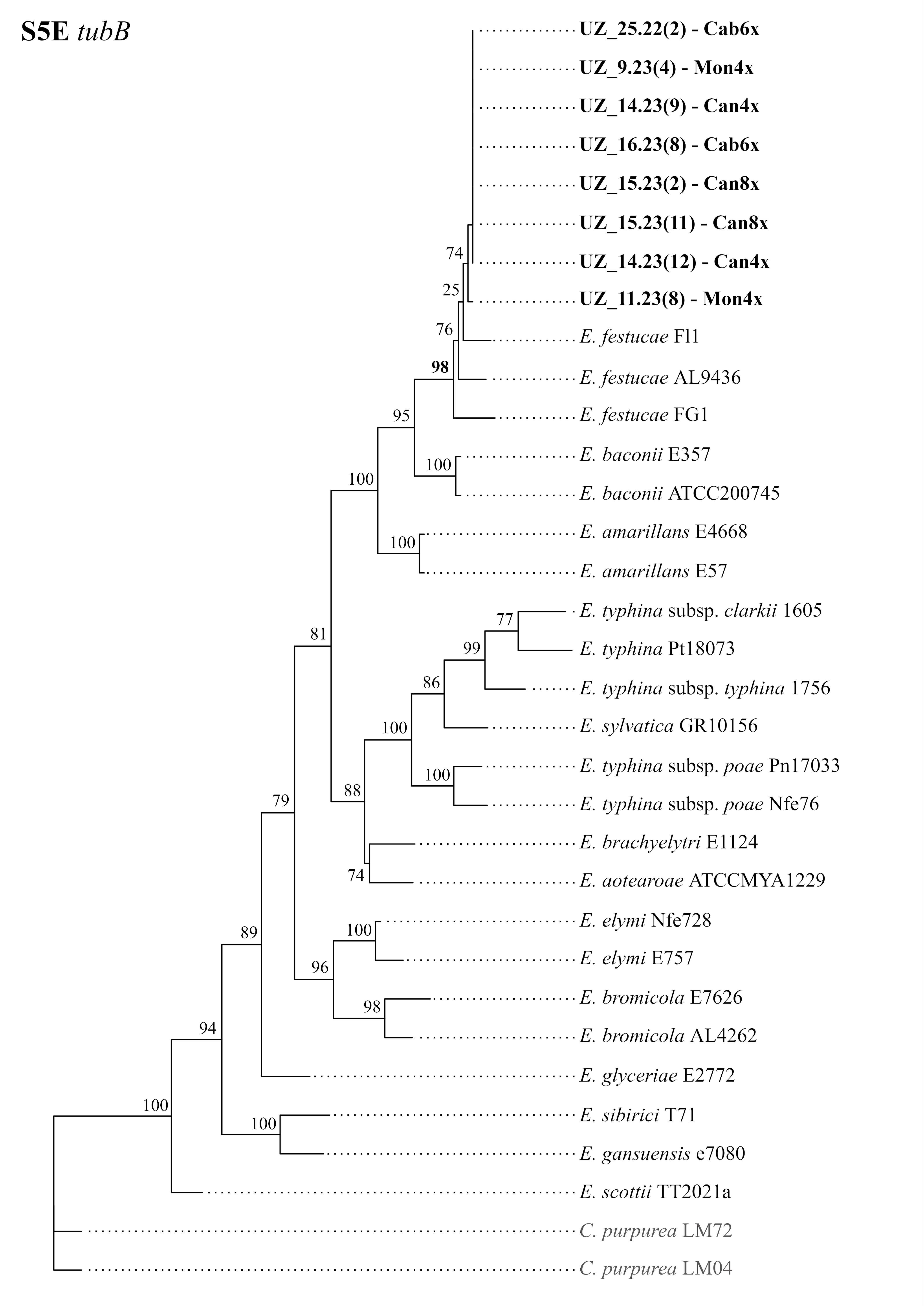


**Figure S5.** Maximum-likelihood phylogenetic trees (IQ-TREE2) of *Epichloë festucae* based on five nuclear loci: (A) γ-actin (*actG*), (B) calmodulin (*CalM*), (C) ITS region, (D) translation elongation factor 1-α (*tefA*), and (E) β-tubulin (*tubB*). Newly analyzed samples (Table S12) are in bold; sequences of other *Epichloë species* and the outgroup *Claviceps purpurea* were obtained from NCBI (Table S3). Node values represent ultrafast bootstrap support.


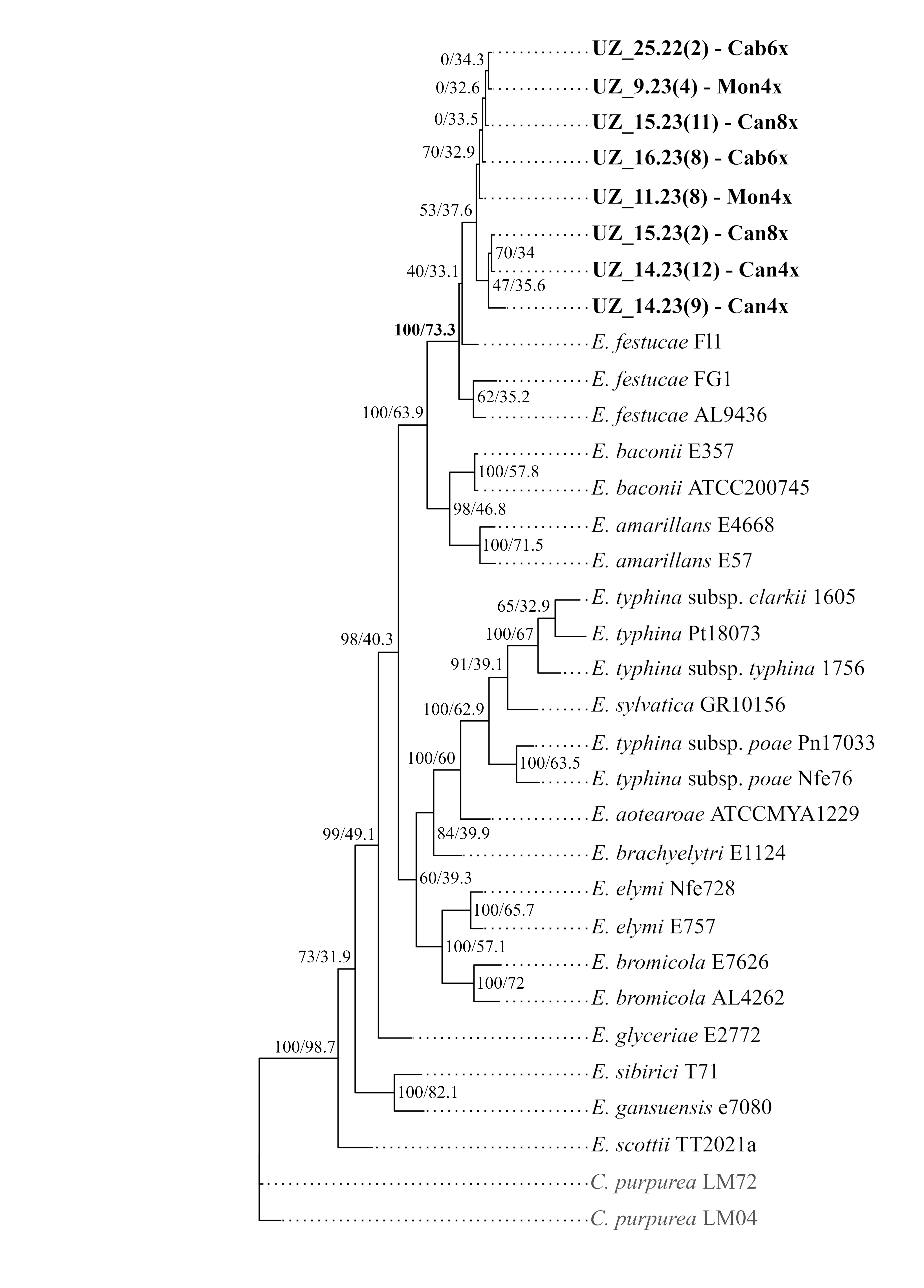


**Figure S6.** Concatenated maximum-likelihood phylogeny of *Epichloë festucae* based on five nuclear loci (*actG*, *CalM*, ITS, *tefA*, and *tubB*), constructed with IQ-TREE2. Newly analyzed samples (Table S12) are in bold; sequences of other *Epichloë species* and the outgroup *Claviceps purpurea* were obtained from NCBI (Table S3). Branch labels show ultrafast bootstrap support (BS) and likelihood‑based site concordance factor (sCFL).

**Table S1**. Ploidy information for 22 pooid host grasses and their *Epichloë* endophytes. Note that host ploidy in the literature is typically measured independently of infection status; thus, cross-taxon correlations between host and endophyte ploidy generally require assuming that cytotypes in infected populations match those reported for the species as a whole. Columns show in order: (A) host taxon; (B) host ploidy and/or chromosome number (x = chromosome base number, “p”x = ploidy level, 2n = chromosome number); (C) method applied for assessment of plant ploidy level (CC = chromosome count, FC = flow cytometry); (D) whether the endophyte is present in the measured host specimens (E+?; Yes/No); (E) plant host reference; (F) endophyte taxon; (G) endophyte ploidy ( H = haploid, DIHET = diheteroploid, TRIHET = triheteroploid); (H) methods applied for assessment of endophyte ploidy level (GA = Genome assembly, M = morphometry, EK = electrophoretic karyotype); (I) hybrid status and reproduction ability (NH = Non-hybrid, DIHET = Diheteroploid, TRIHET = Triheteroploid; Asexual/Sexual); and (J) endophyte references. “(Presumed)” indicates ploidy inferred from genomic data or other methodologies, without direct measurement.

| **(A)**  **Host taxon** | **(B)**  **Host ploidy** | **(C)**  **Method (H)** | **(D)**  **E^+^?** | **(E)**  **Plant Ref.** | **(F)**  **Endoph. taxon** | **(G)**  **Endoph. ploidy** | **(H)**  **Method (E)** | **(I)**  **Hyb. / Repr.** | **(J)**  **Endoph. Ref.** |
| --- | --- | --- | --- | --- | --- | --- | --- | --- | --- |
| *Agrostis capillaris* | 4x (2n=28) | FC | No | Bonos et al. (2002) | *Epichloë baconii* | H (presumed) | GA | NH, Sexual | White (1993)  Quenu et al. (2022) |
| *Brachyelytrum erectum* | 2x (2n=22) | CC | No | Bowden (1960) | *Epichloë brachyelytri* | H (presumed) | M; GA | NH, Sexual | Schardl and Leuchtmann (1999); Quenu et al. (2022) |
| *Brachypodium sylvaticum* | 2x (2n=18) | CC | No | Betekhtin et al. (2014) | *Epichloë sylvatica* | H (presumed) | M; GA | NH, Sexual | Leuchtmann and Schardl (1998); Quenu et al. (2022) |
| *Bromus laevipes* | 2x (2n=14) | CC | No | Halabi et al. (2023) | *Epichloë typhina* subsp. *poae* | H (presumed) | GA | NH, Sexual | Charlton et al. (2014)  Quenu et al. (2022) |
| *Dactylis glomerata* | 2x–6x; 4x (2n=28) | CC + FC | No | Vilhar et al. (2002) | *Epichloë typhina* | H (presumed) | GA | NH, Sexual | Chung and Schardl (1997);  Quenu et al. (2022) |
| *Elymus dahuricus* | 6x (2n=42) | CC | No | Jiang et al. (2023) | *Epichloë bromicola* | H (presumed) | GA | NH, Sexual | Shi et al. (2017);  Leuchtmann and Schardl (1998); Quenu et al. (2022) |
| *Elymus virginicus* | 4x (2n=28) | CC | No | Redmann and Borgaonkar (1966) | *Epichloë elymi* | H (presumed) | GA | NH, Sexual | Schardl and Leuchtmann (1999); Quenu et al. (2022) |
| *Festuca arundinacea* | 6x (2n=42) | CC | No | Kopecký et al. (2008) | *Epichloë coenophiala* | TRIHET | M; EK; GA | H, Asexual | Schardl et al. (2004);  Florea et al. (2021);  Lee et al. (2025) |
| *Festuca ovina s.l.* | 2x–4x | CC, FC | No | Qiu et al. (2020)  Loureiro et al. (2007) | *Epichloë festucae* | H (presumed) | M | NH, Sexual | Leuchtmann et al. (2014); Wäli et al. (2007) |
| *Festuca pratensis* | 2x (2n=14) | CC + FC | No | Ansari et al. (2022) | *Epichloë uncinata* | DIHET | M; GA | H, Asexual | Leuchtmann et al. (2014);  Cagnano et al. (2019) |
| *Festuca rothmaleri* | 4x (2n=28); 6x (2n=42); 8x (2n=56) | CC + FC | **Yes** | **This study** | *Epichloë festucae* | Haploid | FC + M | NH, Asexual | **This study** |
| *Festuca rubra s.l.* | 4x, 6x, 8x | CC; FC | No | Šmarda et al. (2008);  Dirihan et al. (2016) | *Epichloë festucae* | H (presumed) | M | NH, Asexual | Zabalgogeazcoa et al. (1999); Dirihan et al. (2016) |
| *Festuca rubra* subsp. *pruinosa* | 6x (2n=42) | CC | No | Loureiro et al. (2007) | *Epichloë festucae* | H (presumed) | M | NH, Asexual | Zabalgogeazcoa et al. (2006) |
| *Festuca vivipara* | 4x (2n=28) | CC + FC | Yes | Gundel et al. (2014) | *Epichloë festucae* | H (presumed) | M | NH, Asexual | Gundel et al. (2014) |
| *Holcus lanatus* | 2x (2n=14) | CC | No | Carroll and Jones (1962) | *Epichloë typhina* subsp. *clarkii* | H (presumed) | GA | NH, Sexual | Treindl et al. (2021) |
| *Lolium multiflorum* | 2x (2n=14) | CC + FC | No | Inda and Wolny (2013) | *Epichloë occultans* | DIHET | NA | H, Asexual | Moon et al. (2007)  Leuchtmann et al. (2014) |
| *Lolium perenne* | 2x (2n=14) | CC + FC | No | Inda and Wolny (2013) | *Epichloë* sp. (LpTG-2) | DIHET | GA | H, Asexual | Moon et al. (2000);  Hettiarachchige et al. (2015) |
| *Lolium perenne* | 2x (2n=14) | CC + FC | No | Inda and Wolny (2013) | *Epichloë hybrida* | DIHET | GA; EK | H, Asexual | Campbell et al. (2017); Winter et al. (2018); Leuchtmann et al. (2014) |
| *Lolium perenne* | 2x (2n=14) | CC + FC | No | Inda and Wolny (2013) | *Epichloë festucae* var. *lolii* | H (presumed) | GA | NH, Asexual | Hettiarachchige et al. (2015) |
| *Melica uniflora* | 2x (2n=18) | CC | No | Gadella and Kliphuis (1963) | *Epichloë scottii* | H (presumed) | GA | NH, Sexual | Thünen et al. (2022) |
| *Poa nemoralis* | 4x (2n=28); 6x (2n=42); 8x (2n=56) | CC | No | Müntzing (1933) | *Epichloë typhina* subsp. *poae* | Haploid (presumed) | GA | NH, Sexual | Tadych et al. (2012);  Quenu et al. (2022) |
| *Poa trivialis* | 2x (2n=14) | CC | No | Ahmed et al. (1972) | *Epichloë typhina* | Haploid (presumed) | GA | NH, Sexual | Quenu et al. (2022) |

**Table S2.** Information on primers pairs and PCR programs used in the amplification of the five barcoding loci (*actG*, *CalM*, ITS, *tefA*, *tubB*) and both mating type idiomorphs (MAT1-1 and MAT1-2) studied in *Epichloë festucae* samples. References correspond to the origin of the primers and the original PCR programs used. The optimized PCR programs are divided into three main steps: Initial Denaturation (Denat.), PCR cycles (Cycles [Denat. / Anneal. / Exten.]), and Final Extension (Exten.). The PCR master mix was prepared following the recommended protocol for KAPA Taq polymerase (Kapa Biosystems) with minor modifications. PCR products of the five barcoding loci were purified with Illustra^TM^ ExoProStar^TM^ 1-Step (GE Healthcare, Life Sciences) following the manufacturer's instructions and sent to the STAB VIDA facility (Caparica, Portugal) for bidirectional Sanger sequencing.

| **Primers information** | | | **Optimized PCR programs** | | |
| --- | --- | --- | --- | --- | --- |
| **Primers** | **Primer sequence (5'-3')** | **Reference** | **Denat.** | **Cycles [Denat. / Anneal. / Exten.]** | **Exten.** |
| act1-exon1d-1 | TAA TCA GTC ACA TGG AGG GT | (Moon et al. 2002) | 94ºC,  3 min | 40 x [94ºC, 45s / 58 ºC, 45s / 72 ºC, 90s] | 72ºC,  5 min |
| act1-exon6u-1 | AAC CAC CGA TCC AGA CAG AGT |  |  |  |  |
| cal-exon1d | TAT CAA ATT TTC CAC CAT GG | McCargo et al. (2014) | 94ºC,  2 min | 40 x [94ºC, 45s / 50ºC, 45s / 72ºC, 60s] | 72ºC, 10 min |
| cal-exon7u | TAC TTC TGC ATC ATA AGC T |  |  |  |  |
| ITS5 | GGA AGT AAA AGT CGT AAC AAG G | White et al. (1990) | 95ºC, 2 min | 35 x [95ºC, 30s / 56ºC, 30s / 72ºC, 45s] | 72ºC,  5 min |
| ITS4 | TCC TCC GCT TAT TGA TAT GC |  |  |  |  |
| tef1-exon1d | GGG TAA GGA CGA AAA GAC TCA | Gentile et al. (2005) | 94ºC,  3 min | 40 x [94ºC, 45s / 59ºC, 45s / 72ºC, 60s] | 72ºC,  5 min |
| tef1-exon6u | CGG CAG CGA TAA TCA GGA TAG |  |  |  |  |
| tubF | GTT TCG TCC GAG TTC TCG AC | Gundel et al. (2014) |  |  |  |
| tubR | ACC GAG AAA AAT GCG TGA GAT |  |  |  |  |
| mtAC-F | CAA TGG TGG TCA CCT GAGAAG | Florea et al. (2015) | 94ºC,  9 min | 35 x [94ºC, 30s / 59ºC, 30s / 72ºC, 1 min] | 72ºC,  7 min |
| mtAC-R | CGG TCT CAT TCT TCC AGA GAG AGG |  |  |  |  |
| mtBA-F | TCT ACC GCA AGG AAC GAC ACA ATA CCG |  |  |  |  |
| mtBA-R | GCT TTT CCA GCA AGG CTT GCT TGA CTC |  |  |  |  |

**Table S3.** Reference genomes of *Epichloë* spp. and the outgroup (*Claviceps purpurea*) species, obtained from NCBI, whose barcoding sequences of the 5 loci were included in the phylogenetic analysis of the *Epichloë festucae* samples under study, isolated from their *Festuca rothmaleri* host plants. Information on the fungal species, specimen ID, host species, NCBI GeneBank accession, and genome size for each sample is provided.

| **Species** | ***Epichloë* specimen ID** | **Host species** | **NCBI Accession** | **Reference** | **Size (Mb)** |
| --- | --- | --- | --- | --- | --- |
| *Claviceps purpurea* | C.purpurea_LM04 | Tricosecale | [GCA_029405315.1](https://www.ncbi.nlm.nih.gov/datasets/genome/GCA_029405315.1/) | Witte et al. (2023) | 33.2 |
| *Claviceps purpurea* | C.purpurea_LM72 | *Alopecurus myosuroides* | [GCA_029405325.1](https://www.ncbi.nlm.nih.gov/datasets/genome/GCA_029405325.1/) | Witte et al. (2023) | 34.2 |
| *Epichloë amarillans* | E.amarillans_E57 | NA | [GCA_000223075.2](https://www.ncbi.nlm.nih.gov/datasets/genome/GCA_000223075.2/) | NA | 38.1 |
| *Epichloë amarillans* | E.amarillans_E4668 | NA | [GCA_000877375.1](https://www.ncbi.nlm.nih.gov/datasets/genome/GCA_000877375.1/) | NA | 40.9 |
| *Epichloë aotearoae* | E.aotearoae_ATCCMYA1229 | *Echinopogon ovatus* | [GCA_000729855.1](https://www.ncbi.nlm.nih.gov/datasets/genome/GCA_000729855.1/) | NA | 34.4 |
| *Epichloë baconii* | E.baconii_ATCC200745 | *Calamagrostis villosa* | [GCA_000729845.1](https://www.ncbi.nlm.nih.gov/datasets/genome/GCA_000729845.1/) | NA | 38.1 |
| *Epichloë baconii* | E.baconii_E357 | *Calamagrostis villosa* | [GCA_023650635.1](https://www.ncbi.nlm.nih.gov/datasets/genome/GCA_023650635.1/) | Quenu et al. (2022) | 39.2 |
| *Epichloë brachyelytri* | E.brachyelytri_E1124 | *Brachyelytrum erectum* | [GCA_023650705.1](https://www.ncbi.nlm.nih.gov/datasets/genome/GCA_023650705.1/) | Quenu et al. (2022) | 44.6 |
| *Epichloë bromicola* | E.bromicola_AL4262 | *Thinopyrum intermedium* | [GCA_001008055.1](https://www.ncbi.nlm.nih.gov/datasets/genome/GCA_001008055.1/) | NA | 32.9 |
| *Epichloë bromicola* | E.bromicola_E7626 | *Campeiostachys dahurica* | [GCA_002319005.1](https://www.ncbi.nlm.nih.gov/datasets/genome/GCA_002319005.1/) | Shi et al. (2017) | 29.9 |
| *Epichloë elymi* | E.elymi_E757 | *Elymus virginicus* | [GCA_002591845.1](https://www.ncbi.nlm.nih.gov/datasets/genome/GCA_002591845.1/) | NA | 26.3 |
| *Epichloë elymi* | E.elymi_Nfe728 | *Elymus virginicus* | [GCA_023658475.1](https://www.ncbi.nlm.nih.gov/datasets/genome/GCA_023658475.1/) | Quenu et al. (2022) | 34.1 |
| *Epichloë festucae* | E.festucae_AL9436 | *Festuca pratensis* | [GCA_002318955.1](https://www.ncbi.nlm.nih.gov/datasets/genome/GCA_002318955.1/) | NA | 29.3 |
| *Epichloë festucae* | E.festucae_FG1 | *Festuca glauca* | [GCA_002163435.1](https://www.ncbi.nlm.nih.gov/datasets/genome/GCA_002163435.1/) | NA | 35.7 |
| *Epichloë festucae* | E.festucae_Fl1 | *Festuca longifolia* | [GCA_003814445.1](https://www.ncbi.nlm.nih.gov/datasets/genome/GCA_003814445.1/) | Quenu et al. (2022) | 35.0 |
| *Epichloë gansuensis* | E.gansuensis_e7080 | *Achnatherum inebrians* | [GCA_023658505.1](https://www.ncbi.nlm.nih.gov/datasets/genome/GCA_023658505.1/) | Quenu et al. (2022) | 39.5 |
| *Epichloë glyceriae* | E.glyceriae_E2772 | *Glyceria striata* | [GCA_023658535.1](https://www.ncbi.nlm.nih.gov/datasets/genome/GCA_023658535.1/) | Quenu et al. (2022) | 43.0 |
| *Epichloë scottii* | E.scottii_TT2021a | *Melica uniflora* | [GCA_021950295.1](https://www.ncbi.nlm.nih.gov/datasets/genome/GCA_021950295.1/) | Thünen et al. (2022) | 37.2 |
| *Epichloë sibirici* | E.sibirici_T71 | *Stipa sibirica* | [GCA_022702365.1](https://www.ncbi.nlm.nih.gov/datasets/genome/GCA_022702365.1/) | NA | 33.0 |
| *Epichloë sylvatica* | E.sylvatica_GR10156 | *Brachypodium sylvaticum* | [GCA_001008265.1](https://www.ncbi.nlm.nih.gov/datasets/genome/GCA_001008265.1/) | NA | 36.1 |
| *Epichloë typhina* | E.typhina_Pt18073 | *Poa trivialis* | [GCA_023658615.1](https://www.ncbi.nlm.nih.gov/datasets/genome/GCA_023658615.1/) | Quenu et al. (2022) | 39.2 |
| *Epichloë typhina* subsp. *clarkii* | E.typhinaclarkii_1605 | *Holcus lanatus* | [GCA_021378265.1](https://www.ncbi.nlm.nih.gov/datasets/genome/GCA_021378265.1/) | Treindl et al. (2021) | 45.6 |
| *Epichloë typhina* subsp. *poae* | E.typhinapoae_Nfe76 | *Bromus laevipes* | [GCA_030389615.1](https://www.ncbi.nlm.nih.gov/datasets/genome/GCA_030389615.1/) | NA | 38.3 |
| *Epichloë typhina* subsp. *poae* | E.typhinapoae_Pn17033 | *Poa nemoralis* | [GCA_023658565.1](https://www.ncbi.nlm.nih.gov/datasets/genome/GCA_023658565.1/) | Quenu et al. (2022) | 34.1 |
| *Epichloë typhina* subsp. *typhina* | E.typhinatyphina_1756 | *Dactylis glomerata* | [GCA_021378235.1](https://www.ncbi.nlm.nih.gov/datasets/genome/GCA_021378235.1/) | Quenu et al. (2022) | 33.8 |

**Table S4.** Genome size estimations (pg/2C) and chromosome counts for the studied samples of *Festuca rothmaleri* at individual and population levels. The study included tetraploid [Mon4x (A), Can4x (B)], hexaploid [Cab6x (C)] and octoploid [Can8x (D)] individuals. Genome sizes are expressed as mean ± SD values at individual (Ind.) and population (Pop.) level. Chromosome numbers matched the genome size values of each of the three ploidy levels detected in this grass species: **(A)** 2n = 4x = 28, **(B)** 2n= 6x = 42; **(C)** 2n = 8x = 56. n, population size; 2n, chromosome number; px, ploidy level.

| **(A) Sample ID** | | **Ind. (pg/2C)** | **Pop. (pg/2C)** | **2n, px** |
| --- | --- | --- | --- | --- |
| Mon4x  (n=15) | UZ_9.23(1) | 7.89 ± 0.13 | 7.54 ± 0.21 | 2n =  4x = 28 |
|  | UZ_9.23(2) | 7.82 ± 0.05 |  |  |
|  | UZ_9.23(4) | 7.66 ± 0.06 |  |  |
|  | UZ_10.23(3) | 7.70 ± 0.09 |  |  |
|  | UZ_10.23(4) | 7.50 ± 0.26 |  |  |
|  | UZ_11.23(1) | 7.25 ± 0.02 |  |  |
|  | UZ_11.23(2) | 7.45 ± 0.02 |  |  |
|  | UZ_11.23(3) | 7.31 ± 0.07 |  |  |
|  | UZ_11.23(4) | 7.63 ± 0.34 |  |  |
|  | UZ_11.23(5) | 7.18 ± 0.08 |  |  |
|  | UZ_11.23(7) | 7.54 ± 0.01 |  |  |
|  | UZ_11.23(8) | 7.43 ± 0.05 |  |  |
|  | UZ_12.23(1) | 7.58 ± 0.04 |  |  |
|  | UZ_12.23(5) | 7.77 ± 0.06 |  |  |
|  | UZ_12.23(6) | 7.38 ± 0.17 |  |  |

| **(B) Sample ID** | | **Ind. (pg/2C)** | **Pop. (pg/2C)** | **2n, px** |
| --- | --- | --- | --- | --- |
| Can4x  (n=15) | UZ_14.23(1) | 7.70 ± 0.09 | 7.61 ± 0.13 | 2n =  4x = 28 |
|  | UZ_14.23(2) | 7.48 ± 0.11 |  |  |
|  | UZ_14.23(3) | 7.45 ± 0.00 |  |  |
|  | UZ_14.23(4) | 7.90 ± 0.02 |  |  |
|  | UZ_14.23(5) | 7.65 ± 0.04 |  |  |
|  | UZ_14.23(6) | 7.73 ± 0.11 |  |  |
|  | UZ_14.23(7) | 7.46 ± 0.07 |  |  |
|  | UZ_14.23(8) | 7.62 ± 0.02 |  |  |
|  | UZ_14.23(9) | 7.57 ± 0.04 |  |  |
|  | UZ_14.23(10) | 7.50 ± 0.02 |  |  |
|  | UZ_14.23(11) | 7.60 ± 0.06 |  |  |
|  | UZ_14.23(12) | 7.74 ± 0.10 |  |  |
|  | UZ_14.23(13) | 7.55 ± 0.03 |  |  |
|  | UZ_14.23(14) | 7.47 ± 0.02 |  |  |
|  | UZ_14.23(15) | 7.65 ± 0.08 |  |  |

| **(C) Sample ID** | | **Ind. (pg/2C)** | **Pop. (pg/2C)** | **2n, px** |
| --- | --- | --- | --- | --- |
| Cab6x  (n=15) | UZ_16.23(1) | 13.14 ± 0.11 | 12.81 ± 0.16 | 2n =  6x = 42 |
|  | UZ_16.23(2) | 12.79 ± 0.03 |  |  |
|  | UZ_16.23(3) | 12.95 ± 0.06 |  |  |
|  | UZ_16.23(4) | 12.81 ± 0.05 |  |  |
|  | UZ_16.23(5) | 12.83 ± 0.06 |  |  |
|  | UZ_16.23(7) | 12.76 ± 0.02 |  |  |
|  | UZ_16.23(8) | 12.78 ± 0.06 |  |  |
|  | UZ_25.22(1) | 12.93 ± 0.07 |  |  |
|  | UZ_25.22(2) | 12.87 ± 0.09 |  |  |
|  | UZ_25.22(3) | 12.61 ± 0.21 |  |  |
|  | UZ_25.23(5) | 12.81 ± 0.03 |  |  |
|  | UZ_25.22(6) | 12.47 ± 0.04 |  |  |
|  | UZ_25.22(13) | 12.87 ± 0.04 |  |  |
|  | UZ_25.22(14) | 12.98 ± 0.06 |  |  |
|  | UZ_25.22(15) | 12.62 ± 0.24 |  |  |

| **(D) Sample ID** | | **Ind. (pg/2C)** | **Pop. (pg/2C)** | **2n, px** |
| --- | --- | --- | --- | --- |
| Can8x  (n=11***)*** | UZ_15.23(2) | 15.73 ± 0.04 | 15.58 ± 0.28 | 2n =  8x = 56 |
|  | UZ_15.23(3) | 15.18 ± 0.07 |  |  |
|  | UZ_15.23(4) | 15.44 ± 0.21 |  |  |
|  | UZ_15.23(5) | 15.99 ± 0.11 |  |  |
|  | UZ_15.23(7) | 15.51 ± 0.07 |  |  |
|  | UZ_15.23(8) | 15.58 ± 0.02 |  |  |
|  | UZ_15.23(10) | 15.43 ± 0.01 |  |  |
|  | UZ_15.23(11) | 16.06 ± 0.05 |  |  |
|  | UZ_15.23(12) | 15.61 ± 0.06 |  |  |
|  | UZ_26.22(1) | 15.23 ± 0.01 |  |  |
|  | UZ_26.22(2) | 15.62 ± 0.16 |  |  |

**Table S5.** PCR-based exploratory screening of mating types MAT1 (785 bp) and MAT2 (215 bp) in *Epichloë* isolates from the three sampling populations (Mon, Cab, Can), using primers listed in Table S2. For this exploratory screening, a sufficient number of individuals from each population were tested to ensure that both mating types could be detected at least once.

| ***Festuca-Epichloë* holobiont specimen** | **Host cytotype / source population** | ***Epichloë* MAT-1** | ***Epichloë* MAT-2** |
| --- | --- | --- | --- |
| UZ_10.23(4) | Mon4x | - | + |
| UZ_11.23(9) | Mon | - | + |
| UZ_12.23(2) | Mon | + | - |
| UZ_12.23(5) | Mon4x | + | - |
| UZ_35.22(1) | Mon | - | + |
| UZ_16.23(1) | Cab6x | + | - |
| UZ_16.23(8) | Cab6x | + | - |
| UZ_25.22(4) | Cab | - | + |
| UZ_25.22(13) | Cab6x | + | - |
| UZ_14.23(1) | Can4x | + | - |
| UZ_14.23(9) | Can4x | + | - |
| UZ_14.23(15) | Can4x | + | - |
| UZ_15.23(10) | Can8x | - | + |
| UZ_15.23(11) | Can8x | - | + |
| UZ_15.23(12) | Can8x | - | + |

**Table S6**. Morphological analysis of asexual characters (conidia, conidiophores) of *Epichloë* *festucae* isolates from host plants of each of the four *Festuca rothmaleri* populations and host ploidy levels under study (labelled here as “Pop”; Mon4x, Can4x, Cab6x, Can8x). Measurements include length of conidia (conidL), width of conidia (conidW), area of conidia (conidA), length of conidiophores (conidophL) and width at the base of conidiophores (conidophW). Values correspond to mean ± SD at individual and population levels [Minimum value**–**Maximum value]. For each character, group measurements with different letters are significantly different based on pairwise comparisons (p < 0.05).

| **Sample information** | | **Morphological characters** | | | | | | | | | |
| --- | --- | --- | --- | --- | --- | --- | --- | --- | --- | --- | --- |
| **Pop** | **Individual** | **conidL (µm)** | | **conidW (µm)** | | **conidA (µm^2^)** | | **conidophL (µm)** | | **conidophW (µm)** | |
| Mon4x | UZ_9.23(4) | 4.3 ± 0.3 [3.4–5.0] | 4.3 ± 0.4  [3.4–5.4]  ^a^ | 2.0 ± 0.2 [1.6–2.3] | 2.0 ± 0.2  [1.6–2.4]  ^a^ | 6.8 ± 0.9 [5.2–8.3] | 6.8 ± 0.9  [5.2–9.4]  ^a^ | 14.1 ± 2.4 [9.6–18.7] | 13.1 ± 2.2 [9.0–18.7]  ^a^ | 1.5 ± 0.2 [1.0–2.1] | 1.8 ± 0.3  [1.0–2.6]  ^a^ |
|  | UZ_10.23(3) | 4.1 ± 0.4 [3.4–5.0] |  | 2.1 ± 0.1 [1.8–2.3] |  | 6.6 ± 0.9 [5.3–8.4] |  | 13.3 ± 2.1 [9.6–18.1] |  | 1.8 ± 0.3 [1.2–2.5] |  |
|  | UZ_10.23(4) | 4.1 ± 0.3 [3.6–5.0] |  | 2.1 ± 0.2 [1.7–2.3] |  | 6.5 ± 0.7 [5.4–8.3] |  | 13.3 ± 2.1 [9.0–17.7] |  | 1.9 ± 0.3 [1.4–2.3] |  |
|  | UZ_12.23(5) | 4.6 ± 0.4 [3.8–5.4] |  | 2.0 ± 0.2 [1.7–2.4] |  | 7.5 ± 1.0 [5.3–9.4] |  | 11.9 ± 1.8 [9.4–15.7] |  | 1.8 ± 0.4 [1.1–2.6] |  |
| Can4x | UZ_14.23(6) | 4.6 ± 0.4 [3.8–5.4] | 4.6 ± 0.4  [3.7–5.4]  ^b^ | 2.0 ± 0.2 [1.5–2.3] | 2.1 ± 0.2  [1.5–2.6]  ^a^ | 7.3 ± 0.9 [5.4–8.8] | 7.3 ± 1.0  [4.8–9.8]  ^b^ | 12.6 ± 2.1 [9.6–16.8] | 12.7 ± 2.0 [8.8–18.6]  ^a^ | 1.8 ± 0.5 [1.0–2.9] | 1.8 ± 0.4  [1.0–2.9]  ^a^ |
|  | UZ_14.23(8) | 4.6 ± 0.4 [3.9–5.4] |  | 2.1 ± 0.2 [1.7–2.6] |  | 7.4 ± 1.0 [6.0–9.8] |  | 13.3 ± 1.8 [10.1–17.0] |  | 1.7 ± 0.4 [1.2–2.6] |  |
|  | UZ_14.23(9) | 4.8 ± 0.4 [3.7–5.4] |  | 2.1 ± 0.2 [1.6–2.4] |  | 7.7 ± 0.9 [6.2–9.4] |  | 12.5 ± 2.0 [9.3–17.6] |  | 1.9 ± 0.4 [1.2 – 2.7] |  |
|  | UZ_14.23(12) | 4.4 ± 0.4 [3.7–5.2] |  | 2.0 ± 0.2 [1.7–2.4] |  | 7.0 ± 1.1 [4.8–8.5] |  | 12.5 ± 2.2 [8.8–18.6] |  | 1.8 ± 0.4 [1.2–2.6] |  |
| Cab6x | UZ_16.23(1) | 4.1 ± 0.3 [3.4–4.9] | 4.3 ± 0.4  [3.4–5.3]  ^a^ | 1.9 ± 0.2 [1.6–2.4] | 2.0 ± 0.2  [1.5–2.4]  ^b^ | 6.3 ± 0.7 [5.1–7.8] | 6.8 ± 0.9  [5.0–8.7]  ^a^ | 10.9 ± 1.5 [7.1–14.7] | 11.8 ± 2.0 [7.1–17.2]  ^b^ | 1.8 ± 0.4 [1.2–2.8] | 1.9 ± 0.4  [1.1–2.9]  ^a^ |
|  | UZ_16.23(5) | 4.3 ± 0.3 [4.0–5.1] |  | 1.9 ± 0.2 [1.5–2.4] |  | 6.8 ± 0.7 [5.5–8.2] |  | 12.3 ± 1.9 [9.0–17.2] |  | 1.9 ± 0.5 [1.1–2.9] |  |
|  | UZ_16.23(8) | 4.4 ± 0.4 [3.6–5.3] |  | 2.0 ± 0.2 [1.5–2.3] |  | 6.6 ± 1.1 [5.0–8.7] |  | 11.5 ± 2.0 [8.3–17.2] |  | 1.8 ± 0.4 [1.2–2.6] |  |
|  | UZ_25.22(4) | 4.6 ± 0.3 [3.9–5.2] |  | 2.0 ± 0.2 [1.6–2.3] |  | 7.4 ± 0.7 [5.6–8.6] |  | 12.3 ± 2.3 [8.2–16.5] |  | 2.0 ± 0.4 [1.3–2.8] |  |
| Can8x | UZ_15.23(2) | 4.4 ± 0.3 [3.7–4.8] | 4.5 ± 0.3  [3.7–5.5]  ^a^ | 2.0 ± 0.2 [1.6–2.4] | 2.0 ± 0.2  [1.6–2.5]  ^a^ | 7.2 ± 0.9 [5.4–8.8] | 7.3 ± 1.0  [4.9–9.7]  ^b^ | 11.9 ± 2.1 [8.8–16.5] | 11.9 ± 2.4 [5.7–18.1]  ^b^ | 1.8 ± 0.4 [1.2–2.5] | 1.8 ± 0.4  [1.2–2.9]  ^a^ |
|  | UZ_15.23(5) | 4.5 ± 0.3 [3.8–5.1] |  | 2.1 ± 0.2 [1.7–2.4] |  | 7.7 ± 1.0 [5.6–9.7] |  | 13.0 ± 2.7 [7.7–18.1] |  | 1.8 ± 0.4 [1.2–2.5] |  |
|  | UZ_15.23(10) | 4.6 ± 0.3 [4.1–5.5] |  | 2.0 ± 0.2 [1.7–2.3] |  | 7.3 ± 1.0 [5.4–9.6] |  | 11.8 ± 2.4 [8.6–16.6] |  | 1.8 ± 0.4 [1.2–2.9] |  |
|  | UZ_15.23(11) | 4.4 ± 0.4 [3.8–5.2] |  | 2.1 ± 0.2 [1.6–2.5] |  | 7.1 ± 0.9 [4.9–9.0] |  | 11.0 ± 2.2 [5.7–15.5] |  | 1.8 ± 0.4 [1.2–2.5] |  |

**Table S7.** Summary of PERMANOVA and variance homogeneity (betadisper) tests for each grouping factor (individual, population of origin, host ploidy level, and the interaction between population and host ploidy level) of the morphological characters of *Epichloë festucae* studied. PERMANOVA results include degrees of freedom (df), proportion of explained variance (R²), F-statistic (F), and associated p-values based on 10,000 permutations. The variance homogeneity test assesses differences in group dispersions (centroid variability) for each factor. Significant p-values (p < 0.05) are marked with an asterisk (*).

| **Predictor** | **PERMANOVA** | | | | **Variance homogenity test** | | |
| --- | --- | --- | --- | --- | --- | --- | --- |
|  | **df** | **R^2^** | **F** | **p-value** | **df** | **F** | **p-value** |
| Individual | 15 | 0.150 | 5.331 | <0.001 * | 15 | 1.429 | 0.129 |
| Population | 2 | 0.051 | 12.555 | <0.001 * | 2 | 2.433 | 0.089 |
| Ploidy | 2 | 0.053 | 13.077 | <0.001 * | 2 | 3.661 | 0.027 * |
| Pop x Ploidy | 3 | 0.064 | 10.550 | <0.001 * | 3 | 2.534 | 0.057 |

**Table S8.** Results of pairwise permutation tests (10,000 resamples) assessing differences in morphological traits of the *Epichloë festucae* samples studied considering three grouping factors. Each table shows the comparison for a given trait, the raw p-value, and the Holm-adjusted p-value to correct for multiple testing. (A) Pairwise comparisons between populations Montemayor del Río, Candelario and El Cabaco (Mon, Can, Cab); (B) Pairwise comparisons between host ploidy levels (4x, 6x, 8x), and (C) Pairwise comparisons considering the interaction between population of origin and host ploidy levels (Mon4x, Can4x, Cab6x, Can8x). Significant adjusted p-values (p < 0.05) are marked with an asterisk (*).

| **(A)** | **Trait** | **Comparison** | **p-value** | **Adj. p-value** |
| --- | --- | --- | --- | --- |
| conidW | | Mon – Can | 0.290 | 0.900 |
|  |  | Mon – Cab | <0.001 * | 0.000 * |
|  |  | Can – Cab | <0.001 * | 0.000 * |
| conidL | | Mon – Can | <0.001 * | 0.000 * |
|  |  | Mon – Cab | 0.225 | 0.900 |
|  |  | Can – Cab | <0.001 * | 0.000 * |
| conidA | | Mon – Can | <0.001 * | 0.000 * |
|  |  | Mon – Cab | 0.540 | 1.000 |
|  |  | Can – Cab | <0.001 * | 0.000 * |
| conidiophW | | Mon – Can | 0.135 | 0.675 |
|  |  | Mon – Cab | 0.055 | 0.330 |
|  |  | Can – Cab | 0.520 | 1.000 |
| conidiophL | | Mon – Can | 0.001 * | 0.008 * |
|  |  | Mon – Cab | <0.001 * | 0.000 * |
|  |  | Can – Cab | 0.031 * | 0.217 |

| **(B)** | **Trait** | **Comparison** | **p-value** | **Adj. p-value** |
| --- | --- | --- | --- | --- |
| conidW | | 4x – 6x | <0.001 * | 0.000 * |
|  |  | 4x – 8x | 0.975 | 1.000 |
|  |  | 6x – 8x | <0.001 * | 0.000 * |
| conidL | | 4x – 6x | 0.043 * | 0.344 |
|  |  | 4x – 8x | 0.488 | 1.000 |
|  |  | 6x – 8x | 0.007 * | 0.063 |
| conidA | | 4x – 6x | 0.003 * | 0.030 * |
|  |  | 4x – 8x | 0.055 | 0.385 |
|  |  | 6x – 8x | <0.001 * | 0.000 * |
| conidiophW | | 4x – 6x | 0.163 | 0.978 |
|  |  | 4x – 8x | 0.400 | 1.000 |
|  |  | 6x – 8x | 0.604 | 1.000 |
| conidiophL | | 4x – 6x | <0.001 * | 0.000 * |
|  |  | 4x – 8x | <0.001 * | 0.000 * |
|  |  | 6x – 8x | 0.613 | 1.000 |

| **(C)** | **Trait** | **Comparison** | **p-value** | **Adj. p-value** |
| --- | --- | --- | --- | --- |
| conidW | | Mon4x – Can4x | 0.207 | 1.000 |
|  |  | Mon4x – Cab6x | 0.001 * | 0.018 * |
|  |  | Mon4x – Can8x | 0.543 | 1.000 |
|  |  | Can4x – Cab6x | <0.001 * | 0.000 * |
|  |  | Can4x – Can8x | 0.540 | 1.000 |
|  |  | Cab6x – Can8x | <0.001 * | 0.000 * |
| conidL | | Mon4x – Can4x | <0.001 * | 0.000 * |
|  |  | Mon4x – Cab6x | 0.226 | 1.000 |
|  |  | Mon4x – Can8x | <0.001 * | 0.000 * |
|  |  | Can4x – Cab6x | <0.001 * | 0.000 * |
|  |  | Can4x – Can8x | 0.011 * | 0.165 |
|  |  | Cab6x – Can8x | 0.005 * | 0.085 |
| conidA | | Mon4x – Can4x | <0.001 * | 0.000 * |
|  |  | Mon4x – Cab6x | 0.541 | 1.000 |
|  |  | Mon4x – Can8x | <0.001 * | 0.000 * |
|  |  | Can4x – Cab6x | <0.001 * | 0.000 * |
|  |  | Can4x – Can8x | 0.784 | 1.000 |
|  |  | Cab6x – Can8x | <0.001 * | 0.000 * |
| conidiophW | | Mon4x – Can4x | 0.225 | 1.000 |
|  |  | Mon4x – Cab6x | 0.054 | 0.756 |
|  |  | Mon4x – Can8x | 0.157 | 1.000 |
|  |  | Can4x – Cab6x | 0.556 | 1.000 |
|  |  | Can4x – Can8x | 0.917 | 1.000 |
|  |  | Cab6x – Can8x | 0.603 | 1.000 |
| conidiophL | | Mon4x – Can4x | 0.111 | 1.000 |
|  |  | Mon4x – Cab6x | <0.001 * | 0.000 * |
|  |  | Mon4x – Can8x | <0.001 * | 0.000 * |
|  |  | Can4x – Cab6x | <0.001 * | 0.000 * |
|  |  | Can4x – Can8x | 0.008 * | 0.128 |
|  |  | Cab6x – Can8x | 0.608 | 1.000 |

**Table S9.** Principal Component Analyses and ANOVAs of morphological traits of *Epichloë festucae* studied across data aggregation levels. (A) Eigenvalues and percentage of variance explained by each of the four principal components (PCs) derived from morphological traits [conidial width (conidW), conidial length (conidL), conidial area (conidA), and conidiophore length (conidiophL)]. PCAs were conducted at two data aggregation levels: mean values per individual (n = 16) and mean values per replicate or plate (n = 48). For each PC, the eigenvalue and the corresponding proportion of explained variance are reported. (B) ANOVA results for linear models testing the effect of population and host ploidy level on PCA scores (PC1 and PC2) and principal component loadings of each morphological trait considered. ANOVAs were conducted at two levels of data aggregation: mean per individual (n = 16) and mean per plate (n = 48). Degrees of freedom (df), F-values (F), and p-values are reported. Statistical significance (p-values < 0.05) is highlighted with an asterisk.

| **(A)** | **Principal**  **Component** | ***Eigenvalue / Explained Variance*** | |
| --- | --- | --- | --- |
|  |  | **Mean per individual**  **(n = 16)** | **Mean per plate**  **(n = 48)** |
| PC1 | | 2.366 / 59.2% | 2.045 / 51.1% |
| PC2 | | 1.096 / 27.4% | 1.050 / 26.3% |
| PC3 | | 0.461 / 11.5% | 0.704 / 17.6% |
| PC4 | | 0.077 / 1.9% | 0.201 / 5.0% |
| Cumulative  (PC1 + PC2) | | 86.6% | 77.4% |

| **(B)** | **Dataset** | **Response** | **ANOVA** | | | | **Principal component loadings** | | | |
| --- | --- | --- | --- | --- | --- | --- | --- | --- | --- | --- |
|  |  |  | **Predictor** | **df** | **F** | **p-value** | **conidA** | **conidL** | **conidW** | **conidiophL** |
| Mean per individual  (n = 16) | | PC1 | Population | 2 | 4.529 | 0.034 * | 0.952 | 0.903 | 0.777 | 0.201 |
|  |  |  | Ploidy | 1 | 0.540 | 0.476 |  |  |  |  |
|  |  |  | Residuals | 12 |  |  |  |  |  |  |
|  |  | PC2 | Population | 2 | 5.529 | 0.020 * | -0.166 | -0.305 | 0.316 | 0.936 |
|  |  |  | Ploidy | 1 | 1.388 | 0.262 |  |  |  |  |
|  |  |  | Residuals | 12 |  |  |  |  |  |  |
| Mean per plate  (n = 48) | | PC1 | Population | 2 | 8.097 | 0.001 * | 0.895 | 0.915 | 0.631 | 0.090 |
|  |  |  | Ploidy | 1 | 0.923 | 0.342 |  |  |  |  |
|  |  |  | Residuals | 44 |  |  |  |  |  |  |
|  |  | PC2 | Population | 2 | 7.332 | 0.002 * | -0.169 | -0.159 | 0.337 | 0.940 |
|  |  |  | Ploidy | 1 | 2.300 | 0.137 |  |  |  |  |
|  |  |  | Residuals | 44 |  |  |  |  |  |  |

**Table S10.**  Performance and discriminant coefficients of Linear Discriminant Analysis (LDA) of morphological traits of *Epichloë festucae* studied by dataset and grouping factor. Model performance metrics (accuracy and Cohen's kappa) for LDA models, including values obtained by leave-one-out cross-validation (LOOCV) and the proportion of traces for the first two discriminant functions (%). In addition, discriminant coefficients (scaling) are provided for each morphological variable, reflecting its contribution to the LD1 and LD2 discriminant functions. Analyses were performed at two levels of data aggregation: mean per individual (n = 16) and mean per plate (n = 48) using three grouping factors: source population, host ploidy level, and their interaction.

| **Dataset** | **Grouping factor** | **LDA classification performance** | | | | | **LDA discriminant coefficients (LD1 \| LD2)** | | | | |
| --- | --- | --- | --- | --- | --- | --- | --- | --- | --- | --- | --- |
|  |  | **Accuracy** | **Kappa** | **LOOCV Accuracy** | **LOOCV Kappa** | **Proportion of trace**  **(LD1 \| LD2)** | **conidW** | **conidL** | **conidA** | **conidiophL** | **conidiophW** |
| Mean per individual  (n = 16) | Population | 0.875 | 0.789 | 0.562 | 0.263 | 70.5% \| 29.5% | 16.286 \| -2.455 | -6.648 \| -2.505 | 3.164 \| -1.189 | 0.188 \| 0.779 | -9.388 \| 0.360 |
|  | Ploidy | 0.812 | 0.692 | 0.688 | 0.459 | 64.9% \| 35.1% | 16.105 \| 3.323 | -7.464 \| 9.559 | -0.173 \| 1.440 | -9.762 \| 2.830 | 4.122 \| -5.284 |
|  | Pop × Ploidy | 0.875 | 0.833 | 0.333 | 0.500 | 53.5% \| 27.4% | -15.216 \| 2.506 | 9.265 \| 8.262 | -4.419 \| -4.905 | 0.122 \| 1.389 | 10.063 \| 2.655 |
| Mean  per plate  (n = 48) | Population | 0.771 | 0.621 | 0.667 | 0.448 | 57.1% \| 42.9% | 2.809 \| -4.351 | -4.642 \| 0.705 | 0.245 \| -1.526 | 0.660 \| -0.351 | 0.305 \| -0.103 |
|  | Ploidy | 0.688 | 0.474 | 0.562 | 0.257 | 69.1% \| 30.9% | -4.729 \| 1.671 | 0.981 \| -3.279 | -0.749 \| 2.427 | -0.759 \| -0.522 | -0.462 \| -0.709 |
|  | Pop × Ploidy | 0.625 | 0.500 | 0.479 | 0.306 | 46.6% \| 38.8% | -2.785 \| 3.891 | 4.582 \| 1.131 | -0.242 \| 0.800 | -0.655 \| 0.488 | -0.303 \| 0.331 |

**Table S11.** Genome size estimates (pg/1C) measured through flow cytometry analysis in *Epichloë festucae* endophytes isolated from host *Festuca rothmaleri* individuals from each population and ploidy group under study (Mon4x, Can4x, Cab6x, Can8x). Measurements were estimated using *Colletotrichum acutatum* strain PT812 as standard. Values were calculated for endophytes isolated from three randomly selected individual plants from each population (Mean ± SD). Four technical replicates were measured for each individual endophyte considered.

| **Sample information** | | **Ind. Genome size (pg/1C)** | **Pop. Genome size (pg/1C)** |
| --- | --- | --- | --- |
| Mon4x | UZ_9.23(4) | 0.042 ± 0.001 | 0.044 ± 0.002 |
|  | UZ_10.23(4) | 0.045 ± 0.002 |  |
|  | UZ_12.23(5) | 0.044 ± 0.002 |  |
| Can4x | UZ_14.23(6) | 0.043 ± 0.001 | 0.045 ± 0.002 |
|  | UZ_14.23(9) | 0.045 ± 0.001 |  |
|  | UZ_14.23(15) | 0.047 ± 0.002 |  |
| Cab6x | UZ_16.23(8) | 0.041 ± 0.001 | 0.045 ± 0.003 |
|  | UZ_25.23(4) | 0.048 ± 0.001 |  |
|  | UZ_25.22(13) | 0.046 ± 0.001 |  |
| Can8x | UZ_15.23(10) | 0.048 ± 0.003 | 0.046 ± 0.002 |
|  | UZ_15.23(11) | 0.045 ± 0.001 |  |
|  | UZ_15.23(12) | 0.045 ± 0.001 |  |

**Table S12.** NCBI GeneBank accession codes for the five nuclear loci [*actG*, *CalM*, ITS, *tefA* (frag1 and frag2), *tubB*] sequenced from *Epichloë festucae* specimens under study isolated from their *Festuca rothmaleri* host plants.

| **Species** | ***Epichloë* specimen ID** | **NCBI GeneBank Accessions** | | | | | |
| --- | --- | --- | --- | --- | --- | --- | --- |
|  |  | ***actG*** | ***CalM*** | **ITS** | ***tefA (*frag1*)*** | ***tefA (*frag2*)*** | ***tubB*** |
| *Epichloë festucae* | UZ_9.23(4)-Mon4x | PV651643 | PV651651 | PV635367 | PV651659 | PV651667 | PV651675 |
|  | UZ_11.23(8)-Mon4x | PV651644 | PV651652 | PV635368 | PV651660 | PV651668 | PV651676 |
|  | UZ_14.23(9)-Can4x | PV651645 | PV651653 | PV635369 | PV651661 | PV651669 | PV651677 |
|  | UZ_14.23(12)-Can4x | PV651646 | PV651654 | PV635370 | PV651662 | PV651670 | PV651678 |
|  | UZ_16.23(8)-Cab6x | PV651649 | PV651657 | PV635373 | PV651665 | PV651673 | PV651681 |
|  | UZ_25.22(2)-Cab6x | PV651650 | PV651658 | PV635374 | PV651666 | PV651674 | PV651682 |
|  | UZ_15.23(2)-Can8x | PV651647 | PV651655 | PV635371 | PV651663 | PV651671 | PV651679 |
|  | UZ_15.23(11)-Can8x | PV651648 | PV651656 | PV635372 | PV651664 | PV651672 | PV651680 |

**Table S13**. Nodal quartet support values for the multispecies coalescent tree of *Epichloë festucae*. Values of q1 (main topology), q2 (first alternative topology), and q3 (second alternative topology) are reported for each node of the species tree inferred using ASTRAL-III.

| **Node** | **q1** | **q2** | **q3** |
| --- | --- | --- | --- |
| 1 | 1 | 0 | 0 |
| 2 | 0.69 | 0.05 | 0.26 |
| 3 | 1 | 0 | 0 |
| 4 | 0.68 | 0.3 | 0.02 |
| 5 | 0.78 | 0.09 | 0.13 |
| 6 | 0.9 | 0.02 | 0.08 |
| 7 | 1 | 0 | 0 |
| 8 | 1 | 0 | 0 |
| 9 | 0.5 | 0.12 | 0.38 |
| 10 | 0.56 | 0.32 | 0.12 |
| 11 | 0.62 | 0 | 0.38 |
| 12 | 0.83 | 0.15 | 0.02 |
| 13 | 1 | 0 | 0 |
| 14 | 0.54 | 0.24 | 0.21 |
| 15 | 1 | 0 | 0 |
| 16 | 0.4 | 0.2 | 0.4 |
| 17 | 1 | 0 | 0 |
| 18 | 0.8 | 0.2 | 0 |
| 19 | 1 | 0 | 0 |
| 20 | 1 | 0 | 0 |
| 21 | 1 | 0 | 0 |
| 22 | 0.53 | 0.02 | 0.44 |
| 23 | 0.4 | 0.3 | 0.3 |
| 24 | 0.67 | 0.18 | 0.14 |
| 25 | 0.6 | 0.14 | 0.27 |
| 26 | 0.55 | 0.32 | 0.12 |
| 27 | 0.54 | 0.16 | 0.3 |
| 28 | 0.49 | 0.21 | 0.3 |
| 29 | 0.38 | 0.33 | 0.3 |
| 30 | 0.44 | 0.2 | 0.35 |

**References Supplementary Material**

Ahmed MK, Jelenkovic G, Dickson WR, Funk^ CR (1972) Chromosome morphology of *Poa Trivialis* L. Canadian Journal of Genetics and Cytology 14: 287–291. https://doi.org/10.1139/g72-036

Ansari HA, Ellison N, Stewart AV, Williams WM (2022) Distribution patterns of rDNA loci in the *Schedonorus*-*Lolium* complex (*Poaceae*). Comparative Cytogenetics 16: 39–54. https://doi.org/10.3897/COMPCYTOGEN.V16.I1.79056

Betekhtin A, Jenkins G, Hasterok R (2014) Reconstructing the evolution of *Brachypodium* genomes using comparative chromosome painting. PLoS ONE 9. https://doi.org/10.1371/journal.pone.0115108

Bonos SA, Plumley KA, Meyer WA (2002) Ploidy determination in *Agrostis* using flow cytometry and morphological traits. Crop Science 42: 192–196.

Bowden WM (1960) Chromosome numbers and taxonomic notes on Northern grasses. Canadian Journal of Botany 38: 117–131. Available from: www.nrcresearchpress.com.

Cagnano G, Roulund N, Jensen CS, Forte FP, Asp T, Leuchtmann A (2019) Large Scale Screening of *Epichloë* endophytes Infecting *Schedonorus pratensis* and Other Forage Grasses Reveals a Relation Between Microsatellite-Based Haplotypes and Loline Alkaloid Levels. Frontiers in Plant Science 10: 10:765. https://doi.org/10.3389/fpls.2019.00765

Campbell MA, Tapper BA, Simpson WR, Johnson RD, Mace W, Ram A, Lukito Y, Dupont PY, Johnson LJ, Scott DB, Ganley ARD, Cox MP (2017) *Epichloë hybrida*, sp. nov., an emerging model system for investigating fungal allopolyploidy. Mycologia 109: 715–729. https://doi.org/10.1080/00275514.2017.1406174

Carroll CP, Jones K (1962) Cytotaxonomic studies in *Holcus*. A morphological study of the triploid f1 hybrid between *Holcus lanatus* L. and *H. mollis* L. New Phytologist 61: 72–84. https://doi.org/10.1111/j.1469-8137.1962.tb06276.x

Charlton ND, Craven KD, Afkhami ME, Hall BA, Ghimire SR, Young CA (2014) Interspecific hybridization and bioactive alkaloid variation increases diversity in endophytic *Epichloë* species of *Bromus laevipes*. FEMS Microbiology Ecology 90: 276–289. https://doi.org/10.1111/1574-6941.12393

Chung KR, Schardl C (1997) Sexual cycle and horizontal transmission of the grass symbiont *Epichloë typhina*. Mycological Research 101: 295–301. https://doi.org/DOI:10.1017/S0953756296003564.

Dirihan S, Helander M, Väre H, Gundel PE, Garibaldi LA, Irisarri JGN, Saloniemi I, Saikkonen K (2016) Geographic variation in *Festuca rubra* L. ploidy levels and systemic fungal endophyte frequencies. PLoS ONE 11. https://doi.org/10.1371/journal.pone.0166264

Florea S, Schardl CL, Hollin W (2015) Detection and isolation of *Epichloë* species, fungal endophytes of grasses. Current Protocols in Microbiology 2015: 19A.1.1-19A.1.24. https://doi.org/10.1002/9780471729259.mc19a01s38

Florea S, Jaromczyk J, Schardl CL (2021) Non-Transgenic CRISPR-Mediated Knockout of Entire Ergot Alkaloid Gene Clusters in Slow-Growing Asexual Polyploid Fungi. Toxins 13.

Gadella TWJ, Kliphuis E (1963) Chromosome numbers of flowering plants in the Netherlands. cta Botanica Neerlandica 12: 195–230.

Gentile A, Rossi MS, Cabral D, Craven KD, Schardl CL (2005) Origin, divergence, and phylogeny of *Epichloë* endophytes of native Argentine grasses. Molecular Phylogenetics and Evolution 35: 196–208. https://doi.org/10.1016/j.ympev.2005.01.008

Gundel PE, Dirihan S, Helander M, Zabalgogeazcoa I, Väre H, Saikkonen K (2014) Systemic fungal endophytes and ploidy level in *Festuca vivipara* populations in North European Islands. Plant Systematics and Evolution 300: 1683–1691. https://doi.org/10.1007/s00606-014-0994-z

Halabi K, Shafir A, Mayrose I (2023) PloiDB: the plant ploidy database. New Phytologist 240: 918–927. https://doi.org/10.1111/nph.19057

Hettiarachchige IK, Ekanayake PN, Mann RC, Guthridge KM, Sawbridge TI, Spangenberg GC, Forster JW (2015) Phylogenomics of asexual *Epichloë* fungal endophytes forming associations with perennial ryegrass. BMC Evolutionary Biology 15. https://doi.org/10.1186/s12862-015-0349-6

Inda LA, Wolny E (2013) Fluorescent in situ hybridization of the ribosomal RNA genes (5S and 35S) in the genus *Lolium*: *Lolium canariense*, the missing link with *Festuca*? Anales del Jardin Botánico de Madrid 70: 97–102. https://doi.org/10.3989/ajbm

Jiang C, Liu X, Yang Z, Li G (2023) Chromosome Rearrangement in *Elymus dahuricus* Revealed by ND-FISH and Oligo-FISH Painting. Plants 12. https://doi.org/10.3390/plants12183268

Kopecký D, Lukaszewski AJ, Doležel J (2008) Cytogenetics of Festulolium (*Festuca* x *Lolium* hybrids). Cytogenetic and Genome Research 120: 370–383. https://doi.org/10.1159/000121086

Lee K, Bentz P, Vaughn J, Missaoui A (2025) Genomic Characterization of Novel Endophyte Strains from Tall Fescue Shows Genome Fragmentation Post-Hybridization. bioRxiv. https://doi.org/10.1101/2025.02.07.637142

Leuchtmann A, Schardl CL (1998) Mating compatibility and phylogenetic relationships among two new species of *Epichloë* and other congeneric European species. Mycological Research 102: 1169–1182. https://doi.org/10.1017/S0953756298006236

Leuchtmann A, Bacon CW, Schardl CL, White JF, Tadych M (2014) Nomenclatural realignment of *Neotyphodium* species with genus *Epichloë*. Mycologia 106: 202–215. https://doi.org/10.3852/13-251.

Loureiro J, Kopecký D, Castro S, Santos C, Silveira P (2007) Flow cytometric and cytogenetic analyses of Iberian Peninsula *Festuca* spp. Plant Systematics and Evolution 269: 89–105. https://doi.org/10.1007/s00606-007-0564-8

McCargo PD, Iannone LJ, Vignale MV, Schardl CL, Rossi MS (2014) Species diversity of *Epichloë* symbiotic with two grasses from southern Argentinean Patagonia. Mycologia 106: 339–352. https://doi.org/10.3852/106.2.339

Moon CD, Scott B, Schardl CL, Christensen MJ (2000) The evolutionary origins of *Epichloë* endophytes from annual ryegrasses. Mycologia 92: 1103–1118. https://doi.org/10.1080/00275514.2000.12061258

Moon CD, Miles CO, Järlfors U, Schardl CL (2002) The evolutionary origins of three new *Neotyphodium* endophyte species from grasses indigenous to the Southern Hemisphere. Mycologia 94: 694–711. https://doi.org/10.1080/15572536.2003.11833197

Moon CD, Guillaumin J-J, Ravel C, Li C, Craven KD, Schardl CL (2007) New *Neotyphodium* endophyte species from the grass tribes *Stipeae* and *Meliceae*. Mycologia 99: 895–905. https://doi.org/10.1080/15572536.2007.11832521

Müntzing A (1933) Apomictic and sexual seed formation in *Poa*. Hereditas 17: 131–154. https://doi.org/10.1111/j.1601-5223.1933.tb02584.x

Qiu Y, Hamernick S, Ortiz JB, Watkins E (2020) DNA content and ploidy estimation of *Festuca ovina* accessions by flow cytometry. Crop Science 60: 2757–2767. https://doi.org/10.1002/csc2.20229

Quenu M, Treindl AD, Lee K, Takemoto D, Thünen T, Ashrafi S, Winter D, Ganley ARD, Leuchtmann A, Young CA, Cox MP (2022) Telomere-to-Telomere Genome Sequences across a Single Genus Reveal Highly Variable Chromosome Rearrangement Rates but Absolute Stasis of Chromosome Number. Journal of Fungi 8. https://doi.org/10.3390/jof8070670

Redmann RE, Borgaonkar DS (1966) A Cytological Study of the *Hordeae* in the Dakotas. Cytologia 31: 213–219.

Schardl CL, Leuchtmann A (1999) Three new species of *Epichloë* symbiotic with North American grasses. Mycologia 91: 95–107. https://doi.org/10.2307/3761196

Schardl CL, Leuchtmann A, Spiering MJ (2004) Symbioses of grasses with seedborne fungal endophytes. Annual Review of Plant Biology 55: 315–340. https://doi.org/10.1146/annurev.arplant.55.031903.141735

Shi C, An S, Yao Z, Young CA, Panaccione DG, Lee ST, Schardl CL, Li C (2017) Toxin-producing *Epichloë bromicola* strains symbiotic with the forage grass *Elymus dahuricus* in China. Mycologia 109: 847–859. https://doi.org/10.1080/00275514.2018.1426941

Šmarda P, Bureš P, Horová L, Foggi B, Rossi G (2008) Genome size and GC content evolution of *Festuca*: Ancestral expansion and subsequent reduction. Annals of Botany 101: 421–433. https://doi.org/10.1093/aob/mcm307

Tadych M, Ambrose K V., Bergen MS, Belanger FC, White JF (2012) Taxonomic placement of *Epichloë poae* sp. nov. and horizontal dissemination to seedlings via conidia. Fungal Diversity 54: 117–131. https://doi.org/10.1007/s13225-012-0170-0

Thünen T, Becker Y, Cox MP, Ashrafi S (2022) *Epichloë scottii* sp. nov., a new endophyte isolated from *Melica uniflora* is the missing ancestor of *Epichloë disjuncta*. IMA Fungus 13. https://doi.org/10.1186/s43008-022-00088-0

Treindl AD, Stapley J, Winter DJ, Cox MP, Leuchtmann A (2021) Chromosome-level genomes provide insights into genome evolution, organization and size in *Epichloë* fungi. Genomics 113: 4267–4275. https://doi.org/10.3929/ethz-b-000516183

Vilhar B, Vidic T, Jogan N, Dermastia M (2002) Genome size and the nucleolar number as estimators of ploidy level in *Dactylis glomerata* in the Slovenian Alps. Plant Systematics and Evolution 234: 1–13. https://doi.org/10.1007/s00606-002-0186-0

Wäli PR, Ahlholm JU, Helander M, Saikkonen K (2007) Occurrence and genetic structure of the systemic grass endophyte *Epichloë festucae* in fine fescue populations. Microbial Ecology 53: 20–29. https://doi.org/10.1007/s00248-006-9076-2

White JF (1993) Endophyte-host associations in grasses. XIX. A systematic study of some sympatric species of *Epichloë* in England. Mycologia 85: 444–455. Available from: http://www.jstor.orgURL:http://www.jstor.org/stable/3760705http://www.jstor.org/stable/3760705?seq=1&cid=pdf-reference#references_tab_contents.

White TJ, Bruns T, Lee S, Taylor J (1990) Amplification and direct sequencing of fungal ribosomal RNA genes for phylogenetics. In: Innis MA, Gelfand DH, Sninsky JJ, White TJ (Eds), PCR Protocols: A Guide to Methods and Applications. Academic Press, San Diego, 315–322.

Winter DJ, Ganley ARD, Young CA, Liachko I, Schardl CL, Dupont PY, Berry D, Ram A, Scott B, Cox MP (2018) Repeat elements organise 3D genome structure and mediate transcription in the filamentous fungus *Epichloë festucae*. PLoS Genetics 14: e1007467. https://doi.org/10.1371/journal.pgen.1007467

Witte TE, Hicks C, Shoukouhi P, Dadej K, Findlay W, Liu M, Overy DP (2023) Chromosome-level draft genome sequences of three isolates of the toxigenic fungus *Claviceps purpurea* showing structural rearrangements. Microbiology Resource Announcements 12. https://doi.org/10.1128/mra.00234-23

Zabalgogeazcoa I, Vázquez De Aldana BR, García Criado B, García Ciudad A (1999) The infection of *Festuca rubra* by the fungal endophyte *Epichloë festucae* in Mediterranean permanent grasslands. Grass and Forage Science 54: 91–95. https://doi.org/10.1046/j.1365-2494.1999.00155.x

Zabalgogeazcoa I, Romo M, Keck E, Vázquez De Aldana BR, García Ciudad A, García Criado B (2006) The infection of *Festuca rubra* subsp. *pruinosa* by *Epichloë festucae*. Grass and Forage Science 61: 71–76. https://doi.org/10.1111/j.1365-2494.2006.00509.x
